# Identification of Functional Differences in Metabolic Networks Using Comparative Genomics and Constraint-Based Models

**DOI:** 10.1371/journal.pone.0034670

**Published:** 2012-04-16

**Authors:** Joshua J. Hamilton, Jennifer L. Reed

**Affiliations:** Department of Chemical and Biological Engineering, University of Wisconsin-Madison, Madison, Wisconsin, United States of America,; King's College London, United Kingdom

## Abstract

Genome-scale network reconstructions are useful tools for understanding cellular metabolism, and comparisons of such reconstructions can provide insight into metabolic differences between organisms. Recent efforts toward comparing genome-scale models have focused primarily on aligning metabolic networks at the reaction level and then looking at differences and similarities in reaction and gene content. However, these reaction comparison approaches are time-consuming and do not identify the effect network differences have on the functional states of the network. We have developed a bilevel mixed-integer programming approach, CONGA, to identify functional differences between metabolic networks by comparing network reconstructions aligned at the gene level. We first identify orthologous genes across two reconstructions and then use CONGA to identify conditions under which differences in gene content give rise to differences in metabolic capabilities. By seeking genes whose deletion in one or both models disproportionately changes flux through a selected reaction (e.g., growth or by-product secretion) in one model over another, we are able to identify structural metabolic network differences enabling unique metabolic capabilities. Using CONGA, we explore functional differences between two metabolic reconstructions of *Escherichia coli* and identify a set of reactions responsible for chemical production differences between the two models. We also use this approach to aid in the development of a genome-scale model of *Synechococcus sp.* PCC 7002. Finally, we propose potential antimicrobial targets in *Mycobacterium tuberculosis* and *Staphylococcus aureus* based on differences in their metabolic capabilities. Through these examples, we demonstrate that a gene-centric approach to comparing metabolic networks allows for a rapid comparison of metabolic models at a functional level. Using CONGA, we can identify differences in reaction and gene content which give rise to different functional predictions. Because CONGA provides a general framework, it can be applied to find functional differences across models and biological systems beyond those presented here.

## Introduction

Advances in genome sequencing and computational modeling techniques have sparked the construction of genome-scale network reconstructions (GENREs) [1] for over 100 prokaryotic and eukaryotic organisms [2]. These reconstructions describe the functions of hundreds of metabolic genes, and enable a concise mathematical representation of an organism's biochemical capabilities via genome-scale models. Constraint-based methods [3] can then be applied to genome-scale models to understand and predict cellular behavior. Genome-scale models are becoming a common framework for representing genomic information, as evidenced by recent works simultaneously reporting genome sequences and metabolic models [4], [5]. Efforts like the new Model SEED database will facilitate this process, by enabling the rapid construction and refinement of network reconstructions as genome annotations change [6].

The abundance of genome sequences has led to advances in comparative genomics, in which biological insight comes from interrogation of genome structure and function across species. The advent of tools such as the Model SEED paves the way for functional comparison of genome-scale reconstructions, but computational methods for comparing models at a functional level have not yet emerged. Existing network comparison approaches such as reconstruction jamborees [7], [8] or metabolic network reconciliation [9] compare models of the same or closely-related organisms with the aim of identifying and reconciling differences between models. These approaches rely on a manual mapping of metabolic compounds and reactions across the networks and then look at differences and similarities in reaction and gene content to identify *structural differences* (e.g., the presence or absence of particular genes or reactions). However, existing approaches do not identify *functional differences* (e.g., differences in organism behavior), or explain how structural differences impact the functional states of the network (e.g., achievable rates of growth or chemical production). Instead, models must be analyzed individually, and a number of simulations may be necessary before functional differences arising from structural differences are observed. Additionally, reaction alignment approaches can be time-consuming, since biochemical databases (such as BiGG, BioCyc, KEGG or SEED [10]–[13]) and model construction platforms (such as Pathway Tools [14] or the Model SEED [6]) may use different nomenclatures or abbreviations to describe metabolites and reactions.

We have developed a bilevel mixed-integer linear programming (MILP) approach to identify functional differences between models by comparing network reconstructions aligned at the gene level, bypassing the need for a time-consuming reaction-level alignment. We call this new constraint-based method CONGA, or **C**omparison **o**f **N**etworks by **G**ene **A**lignment. We first use orthology prediction tools (e.g., bidirectional best-BLAST) to identify sets of orthologs in two organisms based on their genome sequences, and then we use CONGA to identify conditions under which differences in gene content (and thus reaction content) give rise to differences in metabolic capabilities. Because orthologs often encode proteins with the same function, we would expect their gene-protein reaction (GPR) associations, and thus their associated reactions, to be similar. Therefore, a gene-level alignment serves as a proxy for a reaction-level alignment. By identifying genetic perturbation strategies that disproportionately change flux through a selected reaction (e.g., growth or by-product secretion) in one model over another, we are able to functional differences (e.g., biomass yield) between the two organisms. Once these functional differences are found, they can be further evaluated to identify structural differences (e.g., gene and reaction differences) between the organisms' network reconstructions. By using an MILP approach, we are able to identify these differences directly and in an exhaustive fashion, without manually aligning all reactions in the two networks.

We demonstrate that this approach can be used to study both closely- and distantly-related organisms and to address a variety of biological questions, by applying it to three pairs of organisms with increasing phylogenetic distance. We first examine differences between two published metabolic reconstructions of *E. coli* metabolism, *i*JR904 [15] and *i*AF1260 [16]. The *i*AF1260 model is an update to the *i*JR904 model, constructed to more accurately reflect experimental data, including gene essentiality data and growth phenotypes [17], [18]. While both models have been used as tools to help design new chemical production strains [19]–[22], these two models have not been evaluated with respect to differences in their metabolic engineering predictions. By identifying knockout strategies where one model predicts a larger chemical production rate than the other, we are able to determine a small set of reactions responsible for predicted chemical production differences between the two models.

We have also used CONGA to aid in the development of a genome-scale network reconstruction of the photosynthetic cyanobacterium *Synechococcus sp.* PCC 7002, which we name *i*Syp611, by comparing it to the *i*Cce806 reconstruction of *Cyanothece sp.* ATCC 51142 [23]. Photoautotrophic microbes, such as cyanobacteria, possess the ability to fix carbon dioxide and transform light into chemical energy, making them strong candidates for biofuel production hosts [24]–[27]. Through our automated comparison, we also demonstrate the conserved aspects of cyanobacterial physiology, and gain insight into the unique properties of *Synechococcus* and *Cyanothece*.

Finally, we applied CONGA to compare the susceptibility of distantly-related human pathogens to loss of metabolic enzymes. We selected published networks of *M. tuberculosis* H37Rv [28] and *S. aureus* N315 [29] and sought gene knockout strategies that are predicted to be lethal in only one organism. We were then able to identify differences in their metabolic networks which point to unique metabolic functions as possible targets for organism-specific antimicrobials. Such antibiotics are needed to expand the limited scope of existing broad-spectrum antibiotics [30] and to provide novel mechanisms of action which make the transfer of resistance across species less probable [31]–[34]. We show that many of the functions we identified have been experimentally verified as essential, demonstrating that our computational approach allows us to provide a list of candidate enzymes for more focused study. As a component of this comparison, we used three distinct orthology prediction tools to prepare a gene alignment between the pathogens. We then analyzed the number of false positive ortholog calls made by each method, and examined the effect these incorrect orthology assignments had on the results generated by CONGA.

Through these three case studies, we demonstrate that CONGA can be used to rapidly compare metabolic networks regardless of phylogenetic distance. We are also able to show that CONGA has applications in metabolic engineering, model development, and antibiotic discovery. We show that CONGA can facilitate jamboree and network reconciliation efforts by pinpointing those metabolic or genetic differences which give rise to differences in model predictions.

## Results

We have developed a bilevel mixed-integer linear programming (MILP) approach, called CONGA, to identify functional differences between two networks by comparing network reconstructions aligned at the gene level. We have constructed an illustrative example to demonstrate the types of functional differences CONGA can identify. We then present three case studies and demonstrate how CONGA results have implications in metabolic engineering (comparison of *E. coli* models), model development (comparison of cyanobacterial models), and drug discovery (comparison of human pathogen models).

### Identification of Network Differences via CONGA

**Figure 1:**
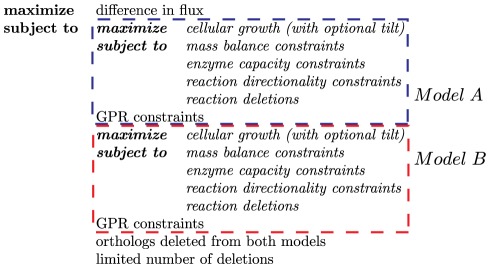
Conceptual structure of the CONGA formulation. CONGA employs a bilevel optimization problem to identify genetic perturbations with nonidentical effects in each of two networks. The outer problem is an MILP which finds gene deletions maximizing the difference in flux value between two reactions in two different models. The inner problems (in italics) are flux-balance analysis (FBA) problems which ensure the flux difference is maximized while both models are maximizing biomass. An optional tilt can be added to the inner problem which forces the flux in the outer problem to the lowest value that still support maximum biomass production. FBA imposes constraints based on reaction stoichiometry, reaction directionality, and enzyme capacities. GPR constraints associate genes to reactions and are used to enforce the reaction deletions associated with the gene deletions in the outer problem. CONGA can select any genes for deletion, with the restriction that orthologous genes present in both models be deleted simultaneously from both models. Finally, a limit may be imposed on the total number of gene deletions.

CONGA identifies functional differences between two networks by comparing network reconstructions aligned at the gene level. The constraint-based method identifies gene deletion strategies leading to different optimal flux distributions in the two networks. CONGA calculates the flux difference between two reactions in different models (e.g., Flux 1 in Species A minus Flux 2 in Species B) and identifies deletions such that the specified flux difference is maximized while both models are simultaneously maximizing biomass (Figure 1).

We refer to a solution identified by CONGA as a *gene deletion set*. CONGA can select any genes for deletion, with the restriction that orthologous genes present in both models be deleted simultaneously from both models. We note that while CONGA can calculate the flux difference between any two reactions, we believe that selecting equivalent reactions (e.g., biomass) provides the most useful objective for comparing models. Via manual investigation of the results, we are able to classify gene deletion sets identified by CONGA as arising due to one of four types of functional network differences:

1. *genetic differences*, in which gene-protein-reaction (GPR) relationships differ between models;
2. *orthology differences*, in which genes encoding enzymes with identical functions cannot be assigned as orthologs (e.g., due to sequence dissimilarity);
3. *metabolic differences*, where one organism has additional reactions which enable it to carry out unique biochemical transformations; and
4. *mixed differences*, which arise due to some combination of types 1–3.

**Figure 2:**
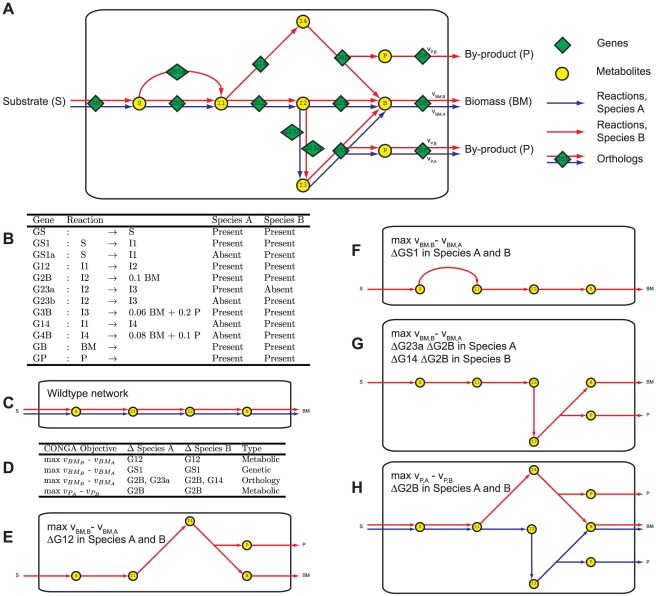
Application of CONGA to an example pair of metabolic networks. (**A**) In these two example networks, substrate (S) is utilized to produce biomass (BM) and some by-product (P). We refer to the two species as A and B, and the biomass- and product-producing reactions as 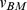 and 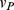, respectively. (**B**) List of genes and reactions present or absent in each network. All shared reactions have orthologs present in both networks, except for the reaction associated with genes G23a and G23b, which are not orthologs. (**C**) A schematic view of the wildtype network behaviors in which flux through 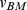 is maximized. (**D**) Gene deletion sets identified by CONGA for the stated CONGA objectives The first three objectives maximize 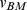 in Species B over Species A. The last objective maximizes 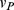 in Species A over Species B. The type of model difference (genetic, orthology, or metabolic) associated with each deletion set is also given. (**E** through **H**) Schematic views of the flux distributions associated with each gene deletion set in **D**. The optimal flux distributions in the example networks change as a result of the gene deletion sets in **D**. Differences in the optimal flux distributions are due to differences in the two networks.

Using two example networks, we demonstrate the types of functional differences CONGA can identify (Figure 2). Each reaction network catalyzes the conversion of substrate (S) to biomass (BM) and some by-product (P) (Figure 2A). We refer to the two species as A and B, and the biomass- and by-product-producing reactions as 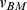 and 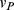, respectively. Each pathway producing biomass gives different yields for BM and P (Figure 2B), though the optimal flux distributions maximizing biomass without any gene deletions are identical in the two organisms (Figure 2C). By applying CONGA with different objective functions, we can identify gene deletion conditions under which network differences become apparent (Figure 2D).

We first used CONGA to compute gene deletion sets maximizing 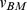 in Species B over Species A (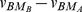). This objective will be greatest when a gene deletion set is predicted to be lethal in Species A and not in Species B. One such deletion set contains the ortholog G12, which is present in both models (Figure 2E). Under this deletion, growth becomes impossible in Species A, whereas Species B has additional reactions which allow it to convert I1 to B via metabolite I4. Thus, this gene deletion set points to a metabolic difference between the two models. CONGA can also be used to identify genetic differences (Figure 2F). For instance, the deletion of GS1 is lethal only in Species A, because Species B has an additional isozyme (GS1a) which carries out the same transformation. Thus, this deletion set points to a genetic difference. Other deletion sets point to orthology differences (Figure 2G). For example, genes G23a and G23b are not orthologs even though they carry out the same reaction. Thus, the deletion of G2B and G23a is lethal in Species A, but Species B can still carry flux through the reaction associated with G23b.

CONGA can also identify how metabolic differences affect cellular phenotypes other than growth rate (Figure 2H). In this example, the objective is to maximize the difference in flux through 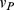 in Species A over Species B (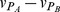). (The resulting phenotypes for each model are analogous to production phenotypes predicted by OptORF [35].) Deleting G2B forces Species A to utilize the lower reaction pathway, producing 0.06 BM and 0.2 P per S. However, the optimal flux distribution for Species B uses the upper reaction pathway, as this route produces more biomass (0.08 BM per S vs 0.06 BM per S via the lower pathway). As a consequence, Species A produces more by-product: 0.2 P per S in Species A vs. 0.1 P per S in Species B.

Because production values may not be unique at the maximum growth rate, CONGA can artificially inflate flux differences between models. This can only occur when the fluxes whose difference is being maximzed (e.g., chemical production rates) differ from the fluxes maximized by each model (e..g, biomass). In this case, we impose a a tilt on the objective of the inner problem. This tilt forces CONGA to identify deletions such that the specified flux difference is maximized when the individual fluxes through each reaction are at their lowest values that still support maximum biomass production. See **Methods** for additional details.

### Comparison of *E. coli* Metabolic Models

We first used CONGA to compare two genome-scale metabolic models of *E. coli*, the *i*JR904 model [15] and the *i*AF1260 model [16]. The *i*AF1260 model extends the *i*JR904 model by compartmentalizing the network (separating the cytoplasm and periplasm), improving the biomass composition, and adding new metabolic reactions. The *i*JR904 model has been used frequently for metabolic engineering studies [36], but to our knowledge no studies have examined the extent to which the *i*AF1260 model's additional metabolic content affects computationally derived strain designs.

To explore the effect of the *i*AF1260 model's larger network, we used CONGA to identify gene deletion strategies for three commonly studied fermentation products–ethanol, lactate, and succinate–seeking identical knockout conditions where the *i*AF1260 model predicted higher production rates than the *i*JR904 model, and vice versa. We refer to such strategies as *model-dominant strategies*. For example, an *i*AF1260-dominant strategy is one in which the same gene deletion set predicts higher chemical production in the *i*AF1260 model than in the *i*JR904 model. Because some of these knockout strategies result in nonunique chemical production rates, model-dominant strategies were identified with respect to the lowest possible production rate consistent with the maximum growth rate.

Our initial CONGA results revealed a need to reconcile the fermentation pathways between the two models, due to changes in representation made in the *i*AF1260 model. We thus modified the *i*JR904 model to reflect these changes and repeated the simulations using the reconciled models. (See Dataset S2 for details.) For ethanol, succinate, and lactate, we identified the top three model-dominant strategies for each model for up to three, four, and five knockouts, respectively. We observed that multiple deletions are necessary to detect differences in production of these latter metabolites, and the difference in yield does not improve significantly beyond four or five knockouts, depending on the model and product. We also employed OptORF [35], without transcriptional regulation, to identify the top three deletion strategies for each model and product, for each number of gene deletions. We refer to these strategies as *OptORF strategies*. These strategies were then compared to the model-dominant strategies identified by CONGA, to determine if optimal OptORF strategies are likely to give similar or different predictions between the two models.

**Figure 3:**
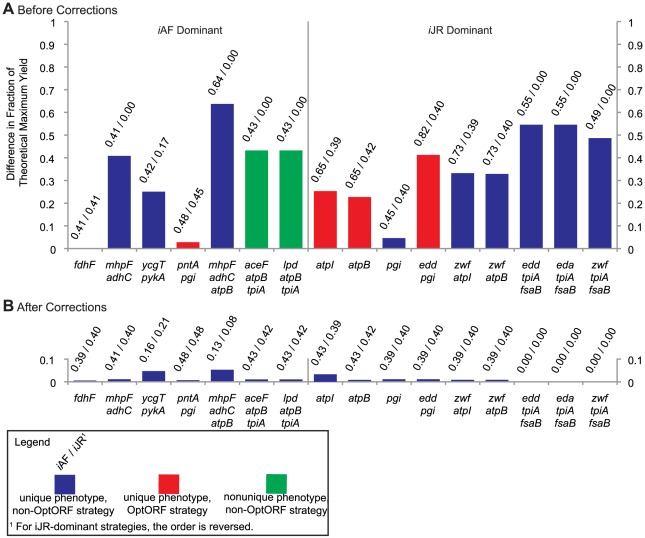
Model-dominant production strategies for ethanol. (**A**) Deletion strategies for ethanol production. Each bar represents the absolute difference in predicted ethanol yields between the *i*JR904 and *i*AF1260 models as a fraction of the maximum theoretical yield (2 ethanol/glucose). Left side: Strategies for which the *i*AF1260 model predicts higher production. Right side: Strategies for which the *i*JR904 model predicts higher production. Corresponding gene deletion strategies involving 1, 2, or 3 genes are given below the figure. Numbers above each bar indicate the fraction of the theoretical maximum yield obtained by each model, with the dominant model listed first. Some strategies have a nonunique ethanol production phenotype, in which multiple ethanol production values can occur at the maximum growth rate. For these scenarios, the production difference calculated by CONGA is from the lowest expected level of ethanol production in each model, and such strategies are indicated in green. Strategies for which the yield of the dominant model meets or exceeds the yield for the third-best OptORF strategy for that model are known as OptORF strategies, and such strategies are indicated in red. (**B**) The same gene deletion strategies after reconciliation of the *i*JR904 and *i*AF1260 networks with respect to metabolic differences.

The CONGA results for the model-dominant strategies for ethanol production are presented in Figure 3A. We observed that only 4 of the 16 (25%) model-dominant strategies were also OptORF strategies (red bars), and none of the triple-deletion model-dominant strategies were OptORF strategies. This suggests that, when examining optimal OptORF strategies for higher numbers of gene knockouts, either model's predictions are likely to be similar at the maximum growth rate. However, the models may predict different ethanol production rates using the same gene deletion set for strategies which do not result in the maximum level of chemical production.

**Figure 4:**
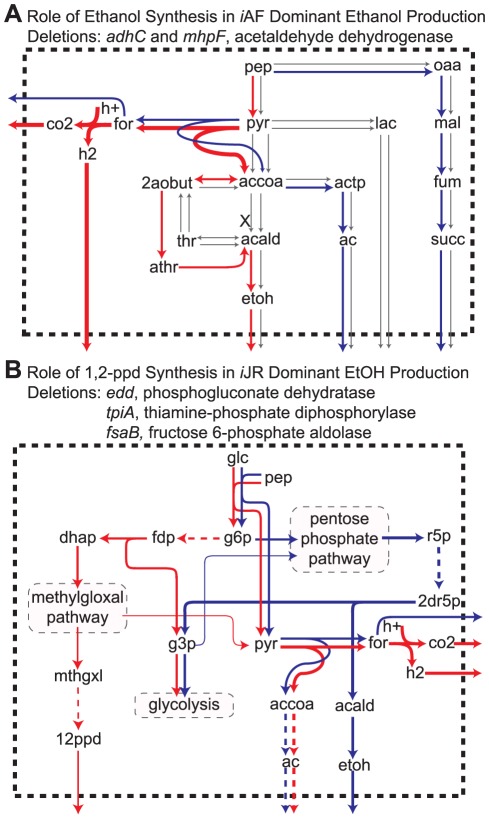
Flux maps illustrating differences in metabolic pathways in *E. coli* GENREs. The text above each map indicates the pathway responsible for the phenotypic difference, the phenotype with which the strategy is associated, and the gene deletion for which the phenotype occurs. (**A**–**B**) Schematic views of the flux distributions associated with the indicated gene deletion set. Metabolites are represented in plain text. Metabolic transformations are indicated via arrows, with thicker arrows indicating higher flux. In some instances, multiple transformations are combined into a single dashed arrow or lumped into a subsystem. Subsystems are indicated by plain text enclosed in a grey rectangle. Fluxes active in the *i*AF1260 network are in red, fluxes active in the *i*JR904 network are in blue, and inactive fluxes are in grey. If gene (reaction) deletions occur in the fermentation pathway, they are indicated by black ‘X’s. Fluxes crossing the dashed boundary indicate transport to the extracellular environment. Metabolite abbreviations: 12 ppd, 1,2-propanediol; 2aobut, L-2-Amino-3-oxobutanoate; actp, acetyl phosphate; athr, allo-threonine. All other abbreviations match those used in the *i*Syp611 metabolic model (see Dataset S2).

The CONGA results for model-dominant strategies for the production of lactate and succinate were quite different (Figure S1A and S2A). Here, 15 of the 30 model-dominant strategies are also OptORF strategies. Of these 15 strategies, 13 are *i*JR904-dominant strategies, with 11 involving the deletion of *mphF* and *adhC* (thereby removing acetaldehyde dehydrogenase). When these two genes are deleted, ethanol synthesis is no longer possible in the *i*JR904 model, while the *i*AF1260 model can synthesize ethanol via a second pathway (Figure 4A). The double deletion of *mphF* and *adhC* enables *i*JR904-dominant strategies for lactate and succinate production, with additional deletions determining whether lactate or succinate is the dominant product. We also observed that the *i*AF1260-dominant strategies for succinate production are all of low-yield (less than 10% the theoretical maximum). In fact, the *i*AF1260 model requires five gene deletions to obtain yields greater than 10% of the theoretical maximum, while the *i*JR904 model requires only two gene deletions. These results demonstrate that CONGA can also be used to identify differences in the ease of coupling growth to chemical production in different models or organisms.

We then set out to investigate which network differences between *i*JR904 and *i*AF1260 account for the production differences associated with each gene deletion set found by CONGA. Of the 46 total model-dominant strategies, 34 (74%) could be attributed to at least one of six metabolic differences between the two models (Table 1). The remaining 12 model-dominant strategies predicted production differences of less than 10% the theoretical maximum yield, and in many cases much less. Two of the network differences (1,2-propanediol synthesis and hexokinase) were associated only with *i*JR904-dominant ethanol production strategies, while others were responsible for more than one set of model-dominant strategies. For example, differences in the succinate transport mechanism were implicated in strategies associated with *i*AF1260-dominant production of ethanol and lactate, and with *i*JR904-dominant production of succinate.

**Table 1:** Explanation of metabolic differences between the *i*JR904 and *i*AF1260 models of *E. coli*.

Metabolic Difference	Description of Metabolic Difference	Functional Effect
1,2-Propanediol Synthesis	The *i*AF1260 model has the ability to secrete 1,2-propanediol; the *i*JR904 model does not.	The ability to convert glucose to 1,2-propanediol gives the *i*AF1260 model greater flexibility in choosing fermentation products under some conditions.
Aldehyde Dehydrogenase	The *i*AF1260 model has a unique aldehyde dehydrogenase which the *i*JR904 model lacks.	This reaction grants the *i*AF1260 model the ability to convert acetaldehyde to acetate using NADP. This reaction was selected for deletion by CONGA in *i*JR dominant strategies, but was never directly implicated in a solution.
Ethanol Synthesis	The *i*AF1260 model has unique reactions to convert acetyl-CoA to acetaldehyde which the *i*JR904 model lacks.	Deletions are possible in which the *i*JR904 model produces no ethanol while the *i*AF1260 model produces ethanol at high levels.
Hexokinase	The *i*AF1260 model has a unique hexokinase that it can use as an alternative to phosphoglucose isomerase (PGI).	The *i*AF1260 model has the ability to recover from multiple-reaction deletions containing PGI, while the *i*JR904 model does not.
Hydrogen Transport	The *i*AF1260 model has the ability to secrete hydrogen gas; the *i*JR904 model does not.	The ability to secrete hydrogen gas allows the *i*AF1260 model to convert formate to 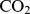 and 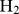 , consuming a proton in the process. This provides the *i*AF1260 model an additional way to consume cytoplasmic 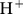 , and changes the preferred fermentation products under some conditions.
Succinate Transport	The *i*AF1260 model employs a hydrogen antiporter for succinate; the *i*JR904 model employs a hydrogen symporter.	Production of succinate becomes less energetically favorable in the *i*AF1260 model, as the synthesis route consumes fewer cytoplasmic protons.

Six metabolic differences accounted for the majority of the model-dominant strategies identified by CONGA.

Many of these network differences affect the balance of possible fermentation products (Figure 4 and Figure S3). For example, the *i*AF1260 network contains an additional pathway to convert acetyl-CoA to ethanol via L-2-amino-3-oxobutanoate and allo-threonine (Figure 4A). As noted above, this extra pathway for ethanol synthesis in the *i*AF1260 model carries flux in many of the *i*JR904-dominant lactate and succinate production strategies, demonstrating that a single network difference can be found under multiple simulation conditions. In other instances, network differences affect flux balances outside the central fermentation pathways (Figure 4B). For example, when the genes *edd* (or *eda*), *tpiA*, and *fsaB* are deleted, disrupting glycolysis and the Entner-Doudoroff pathway, the *i*JR904 and *i*AF1260 models produce different products. The *i*JR904 model converts glucose into ribose-5-phosphate (r5p) via the oxidative and non-oxidative branches of the pentose phosphate pathway. The r5p is then converted to deoxyribose-5-phosphate and broken down into glyceraldehyde-3-phosphate (g3p), which enters glycolysis, and acetaldehyde (acald), which gets converted to ethanol. In contrast, the *i*AF1260 model converts glucose to g3p and dihydroxyacetone phosphate (dhap). As in the *i*JR904 model, g3p enters glycolysis, while dhap enters the methylglyoxal (mthgxl) pathway. Some of the mthgxl is converted to 1,2-propanediol (12 ppd) via a unique 12 ppd synthesis pathway, while the remaining mthgxl continues through the pathway to make pyruvate.

After identifying the metabolic differences that lead to model-dominant strategies, we modified the *i*JR904 and *i*AF1260 networks to contain identical representations of each pathway (Dataset S2) and re-evaluated the phenotype predictions of each knockout strategy. After the network reconciliation, we found that all but one of the knockout mutants are now predicted to have similar production rates (Figure 3B, Figures S1B and S2B).

While other studies have identified functional differences between the *i*JR904 and *i*AF1260 models with respect to growth phenotypes (e.g., gene essentiality predictions [16]) using an enumerative approach, here we compared the two reconstructions with respect to their metabolic engineering predictions using an algorithmic approach that identifies just those conditions resulting in different model predictions. We hypothesized that coupling of metabolites to biomass would be more difficult in the larger *i*AF1260 model, and that the model might have higher production levels (or larger production ranges if multiple products are possible), due to the larger network containing more ways to balance internal fluxes. These hypotheses were not borne out (with the notable exception of coupling succinate production to biomass), as we were able to predict similar production levels using both models. In fact, the production differences we did observe were due to only 21 reactions that represent just 3.5% of the 594 unique metabolic reactions in the *i*AF1260 model (described previously in [16]).

### Cyanobacterial Metabolic Differences

Having analyzed two models of the same organism, we then sought to analyze two models of closely related but distinct organisms, and to examine organisms less well-studied than *E. coli*, to see if CONGA can be used to generate new physiological insights. For this application, we selected two cyanobacteria, *Synechococcus sp.* PCC 7002 and *Cyanothece sp.* ATCC 51142. Very few genome-scale metabolic reconstructions of cyanobacteria have been published to date [37]–[39], and our group has recently developed two more, the *i*Syp611 model of *Synechococcus* (this paper) and the *i*Cce806 model of *Cyanothece* [23]. In order to gain insight into the metabolic similarities and differences between these two cyanobacterial strains, we used CONGA to identify gene deletion sets that were predicted to be lethal in only one cyanobacterial metabolic model, as well as to improve our draft *Synechococcus* reconstruction.

We first applied CONGA to the draft *i*Syp611 model. Some of the gene deletion sets identified by CONGA arose due to missing genes in the draft *i*Syp611 model. For example, CONGA identified gene deletion sets containing protein synthesis enzymes present only in the *i*Cce806 network. *Synechococcus* also has these proteins, but they had not been included in the model. Other network differences arose due to incomplete GPR associations in the draft *i*Syp611 model. For example, the *i*Cce806 model associated HisB with both histidinol-phosphatase and imidazoleglycerol-phosphate dehydratase, while the draft *i*Syp611 network only associated the protein with histidinol-phosphatase. The original annotation indicated the gene was bifunctional, and the draft *i*Syp611 model was updated accordingly. This approach increased the size of the *i*Syp611 model from 542 to 611 genes, an increase in gene content of 13%. This increase in gene content is comparable to that seen in metabolic network reconciliation [9], which was used to expand the gene content of genome-scale models of *Pseudomonas aeruginosa* and *Pseudomonas putida* by 3% and 18%, respectively.

After refining the draft model based on these results, the resulting model (*i*Syp611) was compared again to *i*Cce806 using CONGA. We identified 30 gene deletion sets that are lethal only in the *i*Syp611 model and 36 gene deletion sets that are lethal only in the *i*Cce806 model (Table 2). We found that in many instances different gene deletion sets mapped to the same set of reaction deletions (or *reaction deletion set*). For example, we identified six gene deletion sets lethal in the *i*Syp611 model that all mapped to photosystem II. As a result of these and other redundancies, the 30 gene deletion sets for the *i*Syp611 model reduced to 20 unique reaction deletion sets, and the 36 gene deletion sets for the *i*Cce806 model reduced to 18 unique reaction deletion sets.

**Table 2:** Number of lethal gene deletion sets for the cyanobacterial models *i*Syp611 and *i*Cce806. Numbers in parentheses correspond to unique reaction deletion sets.

	*i*Syp611	*i*Cce804	Interpretation	Example
Genetic	20 (12)	22 (9)	A gene-protein-reaction (GPR) relationship differs between models.	The *i*Syp611 model has a unique isozyme for phosphoglucomutase.
Orthology	4 (4)	4 (4)	Genes encoding enzymes with identical functions cannot be assigned as orthologs.	Both organisms have annotations for dihydroorotase, but the genes are not matched as orthologs due to sequence dissimilarity.
Metabolic	4 (2)	10 (5)	One organism has an additional reaction which enables it to carry out a unique biochemical transformation.	The double deletion of glutamate dehydrogenase and glutamate synthase is lethal only in the *i*Cce806 model.
Mixed	2 (2)	0 (0)	More than one of the above types is implicated in the predicted phenotype difference.	The *Synechococcus* gene for malic enzyme (NADP-catalyzed) is predicted to be an ortholog to the *Cyanothece* gene for malic enzyme (NAD-catalyzed) (orthology difference). The *i*Cce806 has both NAD- and NADP-catalyzed versions of malic enzyme (metabolic difference).
Total	30 (20)	36 (18)		

Functional network differences were classified into one of four types based on their biological interpretation. In many cases, different gene deletion sets led to the same reaction deletion set. The number of unique reaction deletion sets is given in parentheses.

Of the four types of functional network differences, we were most interested in metabolic differences, although the other types are also important. For example, genetic differences may occur because the genes encoding an essential protein have not yet been identified in one organism. In total, the metabolic differences accounted for 4 of 30 gene deletion sets (or 2 of 20 reaction deletion sets) for the *i*Syp611 model and 10 of 36 gene deletion sets (or 5 of 18 reaction deletion sets) for the *i*Cce806 model (Table 3).

**Table 3:** Explanation of metabolic differences between the cyanobacterial models *i*Syp611 (*Synechococcus*) and *i*Cce806 (*Cyanothece*).

Reaction Deletion Set	Lethal In	Explanation of Metabolic Difference
PDH	*i*Syp611	Acetyl-CoA synthase (ACS), pyruvate dehydrogenase (PDH), and phosphotransacetylase (PTA) are responsible for acetyl-CoA synthesis. The *i*Syp611 model requires PDH to supplement the activity of ACS, while the *i*Cce806 model requires PTA. Thus, the deletion of PDH is lethal only in the *i*Syp611 model.
MDH and ME2	*i*Syp611	Fumarate, produced as a byproduct of arginine biosynthesis, is converted to malate and then to oxaloacetate (by malate dehydrogenase, MDH). In the absence of MDH, malic enzyme (ME) can instead convert malate to pyruvate. The *i*Syp611 model contains NADP-catalyzed malic enzyme (ME2), while the *i*Cce806 model contains both NADP- (ME2) and NAD-catalyzed (ME1) malic enzyme. Thus, the deletion of MDH and ME2 is lethal only in the *i*Syp611 model.
ASNS1	*i*Cce806	This reaction synthesizes asparagine. The *i*Syp611 model does not contain this reaction, because *Synechococcus* instead aminates aspartyl-tRNA to asparaginyl-tRNA prior to protein synthesis.
PQPCOR	*i*Cce806	*Cyanothece* is unique among the two cyanobacteria in using plastocyanin during photosynthesis. Hence, the *i*Cce806 model contains the reaction PQPCOR, while the *i*Syp611 model does not.
PTA	*i*Cce806	Acetyl-CoA synthase (ACS), pyruvate dehydrogenase (PDH), and phosphotransacetylase (PTA) are responsible for acetyl-CoA synthesis. The *i*Cce806 model requires PTA to supplement the activity of ACS, while the *i*Syp611 model requires PDH. Thus, the deletion of PTA is lethal only in the *i*Cce806 model.
GLUD and GLUS	*i*Cce806	GLUD (glutamate dehydrogenase) and GLUS (glutamate synthase) synthesize glutamate from alpha-ketoglutarate. This step incorporates ammonia into the metabolism and begins amino acid synthesis. The *i*Syp611 model has an extra reaction, valine-pyruvate aminotransferase (VPAMT), which allows it to recover from this deletion. Under the deletion scenario, ammonia gets combined with pyruvate to make alanine. Alanine is converted to valine which in turn is convered to glutamate.
MDH and PYK	*i*Cce806	Pyruvate synthesis is necessary to meet biomass demands. Pyruvate is normally synthesized from phosphoenolpyruvate via pyruvate kinase (PYK). In the absence of PYK, pyruvate can be synthesized from malate. Malate is produced as a result of biomass demands for arginine and tetrahydrofolate, but in insufficient levels to meet demand. Malate dehydrogenase (MDH) can make up for the demand by converting oxaloacetate to malate. As a consequence, deletion of both genes is lethal. The *i*Syp611 model has the unique reaction aspartase (ASPT), which it can use instead of MDH to convert oxaloacetate to malate, by way of aspartate. As a consequence, MDH function is no longer required in the absence of PYK, and the double deletion is nonlethal.

We identified two unique reaction deletion sets lethal only in the *i*Syp611 model, and five unique reaction deletion sets lethal only in the *i*Cce806 model. From these, we identified seven metabolic differences between the two models.

**Figure 5:**
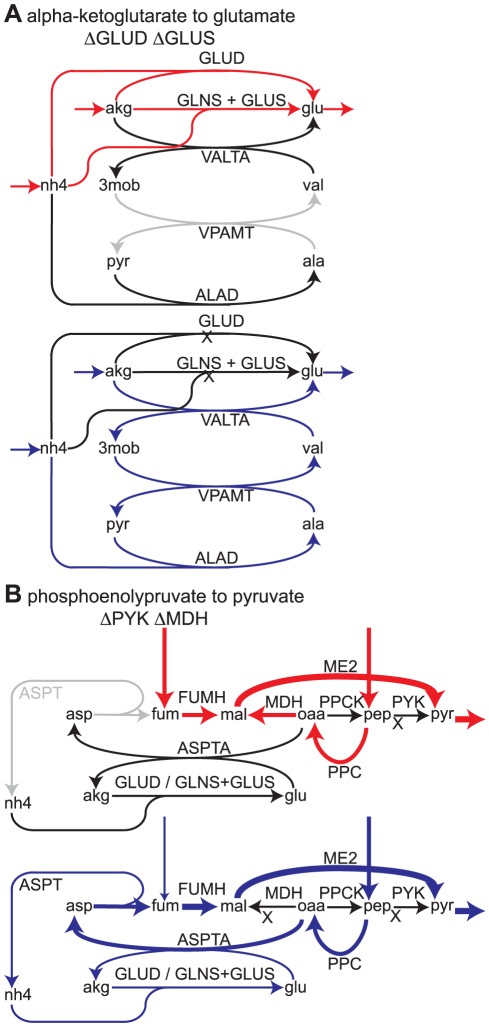
Identified metabolic differences in cyanobacteria. (**A**) Top: Pathways for synthesis of glutamate (glu) from alpha-ketoglutarate (akg) used in *i*Cce806. Bottom: Pathway predicted by the *i*Syp611 model when glutamate dehydrogenase (GLUD) and glutamate synthase (GLUS) are deleted. Valine aminotransferase (VPAMT) enables the synthesis of glutamate from pyruvate (pyr). (**B**) Top: Pathway for conversion of phosphoenolpyruvate (pep) to pyruvate when pyruvate kinase (PYK) is deleted from *i*Cce806. Bottom: Pathway predicted by the *i*Syp611 model when malate dehydrogenase (MDH) is also deleted. Aspartase (ASPT) allows malate (mal) to be synthesized entirely from fumarate (fum), rather than from fumarate and oxaloacetate (oaa). (**A** and **B**) Red arrows indicate flux in the *i*Cce806 model. Blue arrows represent flux in the *i*Syp611 model under the indicated knockout condition. Black arrows indicate inactive reactions and reaction deletions are indicated by black ‘X’s. Gray arrows (top panels) indicate reactions not present in the *i*Cce806 model. Arrow thickness corresponds to relative flux levels. Reaction and metabolite abbreviations are identical in the *i*Syp611 and *i*Cce806 models and are given in Dataset S2.

Two of the reaction deletion sets which are lethal only in the *i*Cce806 model require deletion of two reactions from both models (Figure 5). In the first deletion set (Figure 5A), deletion of glutamate dehydrogenase and glutamate synthase prevents the *i*Cce806 model from synthesizing glutamate. The *i*Syp611 model has a unique reaction, valine amino-transferase (VPAMT), which allows it to recover from this double deletion (blue arrows). In the second deletion set (Figure 5B), deletion of pyruvate kinase and malate dehydrogenase prevents the *i*Cce806 model from making pyruvate. The *i*Syp611 model has another unique reaction, aspartase (ASPT), which enables it to produce pyruvate and recover from the double deletion. A search of the *Cyanothece* genome failed to reveal candidate genes for ASPT and VPAMT, lending support to the hypothesis that they may be true metabolic differences between the two cyanobacteria.

CONGA reveals differences that can be used to reconcile and improve genome-scale metabolic models of closely-related species. We intend to use the remaining genetic and orthology differences found by CONGA as a starting point in further updating our reconstruction, as they may indicate missing or incorrectly annotated genes. CONGA can also identify differences in metabolic capabilities between models: our analysis here indicates *Synechococcus* and *Cyanothece* share a significant number of pathways, with important differences in central and amino acid metabolism.

### Drug Targeting in Human Pathogens

While were able to idenitfy metabolic differences between the two cyanobacteria, many of the differences identified by CONGA were not due to reaction-level differences. We thus sought to use CONGA to explore differences in metabolic capabilities between two dissimilar oganisms, and to exploit those differences to identify organism-specific drug targets. For this application, we applied CONGA to existing models of two phylogenetically distant human pathogens, the *i*NJ661 model of *M. tuberculosis* [28] and the *i*SB619 model of *S. aureus* [29], in order to explore differences in pathogenicity and drug resistance based on differences in reaction and gene content. As with our analysis of the cyanobacterial models, we sought genetic perturbation strategies that were predicted to be lethal in only one organism.

Our preliminary analysis identified a total of 168 unique gene deletion sets, of which 139 (83%) could be traced in whole or in part to genetic or orthology differences. As these differences made up the majority of identified differences, we manually evaluated the quality of the orthology assignments and the original GPR associations. This analysis resulted in the modification of the GPR associations for 19 reactions in the *i*SB619 model and 36 reactions in the *i*NJ661 model (Dataset S2). As a result of these changes, 7 genes were eliminated from and 3 added to the *i*SB619 model, with 10 genes eliminated from and 4 added to the *i*NJ661 model.

**Figure 6:**
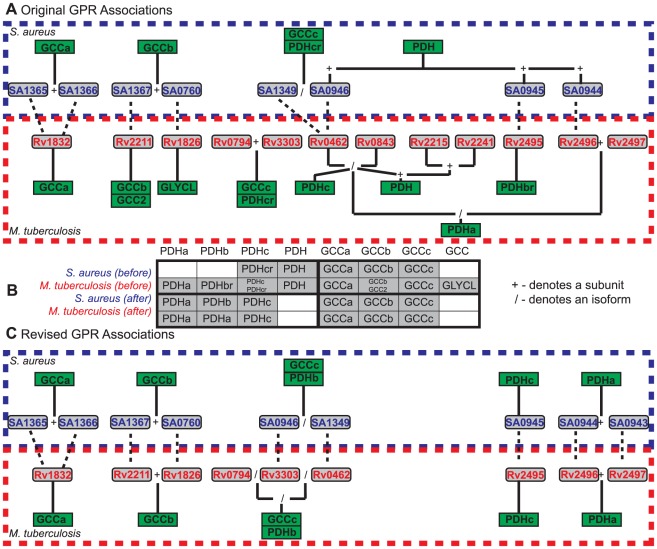
Example adjustment of pathogen models following preliminary analysis. (**A**) Original model annotations for the glycine cleavage (GCC) and pyruvate dehydrogenase (PDH) complexes. Green boxes represent reactions and gray boxes represent genes. *S. aureus* loci are in blue text and *M. tuberculosis* loci are in red text. Dashed lines indicate orthologs and solid lines connect genes to reactions. SA1365 and SA1366 are orthologous to the N-terminus and C-terminus of Rv1832, respectively, and together are orthologous to the entire Rv1832 sequence. A ‘+’ sign between genes indicates a complex; a ‘/’ sign indicates isozymes. (**B**) The two models were inconsistent in their representation of these two enzyme complexes. This table indicates the presence or absence of individual (a, b, c) and lumped (GCC, PDH) reactions before and after model adjustments. The shaded gray boxes indicate the presence of a particular function, and the small black text indicates that model's specific reaction. (**C**) Revised model annotations for the GCC and PDH complexes. The color scheme is the same as in **A**.

A number of these initial genetic- and orthology-related gene deletion sets arose due to different representations of the glycine cleavage complex (GCC) and pyruvate dehydrogenase system (PDH) in the two models (Figure 6A). Both GCC and PDH are composed of three separate enzymes (a, b, and c), each of which carries out a distinct catalytic activity. Deletion of GCC is predicted to be lethal in both organisms, and because one subunit is shared by GCC and PDH, deletions to one complex may affect the other. In its original form, the *i*SB619 reconstruction modeled PDH as an overall reaction, and GCC via its three individual reactions (Figure 6B). In contrast, the *i*NJ661 model represented both PDH and GCC as individual and overall reactions (Figure 6B). Due to these differences, a number of ortholog deletions are lethal in only one model. For example, deletion of the ortholog pair (SA0945, Rv2495) deletes PDH from the *i*SB619 network, but only deletes PDHb from the *i*NJ661 network. The deletion is lethal only in the *i*SB619 model. We thus revised the GPR associations for these complexes to give a consistent representation between the two models (Figure 6C). These changes also required changes to the stoichiometric matrices in each model. (See Dataset S2 for details.)

We applied CONGA again after this initial reconciliation, and identified 71 gene deletion sets lethal only in the *i*SB619 model and 84 gene deletion sets lethal only in the *i*NJ661 model (Table 4). Of these, a total of 99 gene deletion sets (64%) were still due to genetic or orthology differences. Nevertheless, CONGA identified 18 gene deletion sets arising from metabolic differences which were lethal only in the *i*SB619 model, and 38 such gene deletion sets lethal only in the *i*NJ661 model. As with the cyanobacteria, in some instances multiple gene deletion sets mapped to the same reaction deletion set (Table 4). Of these, we examined only those gene deletion sets arising from metabolic differences, and identified 17 unique reaction deletion sets lethal only in the *i*SB619 model and 28 unique reaction deletion sets lethal only in the *i*NJ661 model.

**Table 4:** Number of lethal gene deletion sets for the human pathogen models *i*SB619 and *i*NJ661. Numbers in parentheses correspond to unique reaction deletion sets.

	*i*SB619	*i*NJ661	Interpretation	Example
Genetic	3 (3)	13 (8)	A gene-protein-reaction (GPR) relationship differs between models.	Only the *i*SB619 model has a gene associated with sulfur reductase.
Orthology	17 (17)	14 (14)	Genes encoding enzymes with identical functions can- not be assigned as orthologs.	Both organisms have putative annotations for chorismate mutase, which are not matched as orthologs due to sequence dissimilarity.
Metabolic	18 (17)	38 (28)	One organism has an additional reaction which enables it to carry out a unique biochemical transformation.	The deletion of homoserine kinase is lethal only in the *i*SB619 model.
Mixed	33 (26)	19 (11)	More than one of the above types is implicated in the predicted phenotype difference.	Only the *i*NJ661 model has a gene associated with phosphoserine transaminase (genetic difference). This reaction deletion is nonlethal in the *i*SB619 model because it can utilize alternative pathways to perform this function (metabolic difference).
Total	71 (63)	84 (61)		

Functional network differences were classified into one of four types based on their biological interpretation. In many cases, different gene deletion sets led to the same reaction deletion set. The number of unique reaction deletion sets is given in parentheses.

These 45 unique reaction deletion sets served as the starting set of potential drug targets. We employed a multi-step process to reduce these reaction deletion sets to a set of candidate antibiotic targets. First, because genes may be associated with more than one reaction, we eliminated from each unique reaction deletion set any reactions that were nonessential to the set. For example, CONGA identified the deletion of SA1487 as lethal in *S. aureus*, leading to the reaction deletion set DHFS and THFGLUS. However, the deletion of THFGLUS is not lethal, so THFGLUS was removed from the reaction deletion set, giving the reduced reaction deletion set DHFS. We then examined the reduced reaction deletion sets and eliminated those sets where more than one reaction deletion was required to give a lethal prediction. Such reaction deletion sets are likely to be poor candidates for potential drug targets, because they may require development of a multiple-drug treatment strategy. For example, CONGA identified the reaction deletion set RNDR1, RNDR4 as being lethal in *M. tuberculosis*, with both reaction deletions necessary to give a lethal prediction. This set was subsequently eliminated from the set of candidate antibiotic targets. Finally, we eliminated those reactions included in the Recon 1 genome-scale metabolic model of human metabolism [40], as drugs targeting these reactions may cause adverse side-effects in humans. This procedure yielded 10 reactions as candidate antibiotic targets in *S. aureus* and 37 reactions as candidate antibiotic targets in *M. tuberculosis* (Table 5).

**Table 5:** Potential drug targets in the human pathogens *S. aureus* and *M. tuberculosis*.

Organism	Reaction Deletion Set	Subsystem	Known Drugs
*S. aureus*	ALATA_D	Cell wall synthesis	Vancomycin [41]
*S. aureus*	DHFS	Cofactor synthesis	Trimethoprim and Sulfonamides [48]
*S. aureus*	KAS11 or KAS12 or KAS13	Cell membrane synthesis	Small molecules [45], [46]
*S. aureus*	NNAM	Cofactor synthesis	Small molecules [49]
*S. aureus*	TECA1S or TECA2S or TECA3S or TECA4S	Cell wall synthesis	Vancomycin [41]
*M. tuberculosis*	CHRPL	Cell membrane synthesis	None
*M. tuberculosis*	FACOAL80 or FACOAL160 or FACOAL200 or FACOALPHDCA	Cell wall synthesis	Small molecules [17]
*M. tuberculosis*	FAS80_L or FAS100 or FAS120 or FAS140 or FAS160 or FAS180 or FAS200 or FAS240_L or FAS260 or FASPHDCA	Cell wall synthesis	Pyrazinamide [42]
*M. tuberculosis*	FASm220 or FASm240 or FASm260 or FASm280 or FASm300 or FASm320 or FASm340 or FASm2201 or FASm2202 or FASm2401 or FASm2402 or FASm2601 or FASm2602 or FASm2801 or FASm2802	Cell wall synthesis	Isoniazid [43], [44]
*M. tuberculosis*	MCBTS	Siderophore synthesis	Small molecules [50]
*M. tuberculosis*	PREPPACPH	Cell membrane synthesis	None
*M. tuberculosis*	PPTGS or PPTGS_TB1 or PPTGS_TB1 or UDCPDP	Cell wall synthesis	Ethambutol [43], [44]

We identified five unique reaction deletion sets lethal only in the *i*SB619 model, and seven unique reaction deletion sets lethal only in the *i*NJ661 model. From these, we identified 10 candidate antibiotic targets in *S. aureus* and 37 candidate antibiotic targets in *M. tuberculosis*. Antibiotics targeting some of these reactions have already been developed.

Many of the candidate antibiotic targets are already targeted by existing antibiotics (Table 5), demonstrating that our approach can correctly identify candidate metabolic functions for drug targeting. Most of the reactions for which antimicrobials exist are involved in cell wall and cell membrane synthesis. While both organisms require these biosynthetic capabilities, their cell walls and membranes are structurally different, and so different proteins and reactions are required. These differences are reflected in the standard antimicrobial treatments for these two pathogens. For example, vancomycin binds to the D-alanine terminus of peptidoglycan and prevents the incorporation of teichoic acids into the matrix [41]. Mycobacteria, such as *M. tuberculosis*, have structurally distinct cell walls, for which isoniazid, ethambutol, and pyrazinamide are required treatments [42]–[44]. We were also able to find reports of small molecule inhibitors of fatty acid synthesis in both *S. aureus* [45], [46] and *M. tuberculosis* [47].

We also identified a variety of other metabolic functions which antibiotics do not yet target. For example, the *i*SB619 model requires tetrahydrofuran (THF) and NAD to produce biomass. Unfortunately, many staphylococci are already resistant to inhibitors of THF synthesis [48], while inhibitors of the nicotinamidases *S. aureus* uses for NAD synthesis have only recently been identified [49]. However, *M. tuberculosis* can grow in media lacking THF and NAD [28], suggesting the lack of THF and NAD in the *i*NJ661 biomass equation may reflect a model development choice, rather than a biological difference. We identified *M. tuberculosis*' unique use of siderophores for iron transport, for which biosynthesis inhibitors have been identified [50]. We also identified mycobacteria's use of unique glycolipids, but we were unable to identify inhibitors that have been reported in the literature, making glycolipid synthesis a potential new target for new *M. tuberculosis*-specific antibiotics. Of the remaining organism-specific metabolic functions, two candidate antibiotic targets (nicotinamidase in *S. aureus* and siderophore synthesis in *M. tuberculosis*) had not been identified by previous computational studies of these models [28], [29].

By comparing pathogens against each other, we are able to identify essential functions unique to a particular pathogen. This enables the identification of narrow-spectrum antibiotics tailored to individual pathogens. It is believed that the use of such antibiotics can overcome multi-drug resistance through novel mechanisms of action [32], [33] and slow the rate of resistance transfer across species [31], [34]. We believe our framework provides a rapid means of identifying unique metabolic functions as possible targets for new antimicrobials, and will provide a useful tool for combating the rapid rise of multi-drug resistant bacteria.

### Assessment of Ortholog Calling Methods

**Figure 7:**
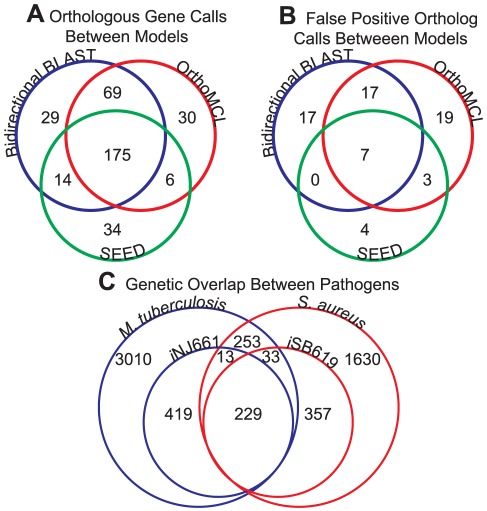
Comparison of ortholog identification methods for *S. aureus* and *M. tuberculosis*. (**A**) Number of model genes identified as orthologs by each of the three methods. Only the orthologs present in both models are included in the diagram. Overlapping areas indicate orthologs identified by one or more methods. (**B**) Number of false positive orthology assignments made by each of the three methods. A false positive orthology assignment indicates the two genes are associated with different reactions in their respective models. Overlapping areas indicate false positives identified by one or more methods. (**C**) Overlap of gene content between the two pathogens, based on SEED FIGfams. Smaller circles represent genetic content of the two models, with the larger circles representing the entire genome. Numbers within overlapping areas indicate numbers of orthologs.

Before CONGA can be applied to a pair of metabolic models, a gene-level alignment must be performed. We perform this alignment by identifying the orthologous genes between the two models, and we force CONGA to select ortholog pairs as a single unit. Prior to applying CONGA to the pathogen models, we examined three methods for identifying orthologous genes (Figure 7). The first method utilized a BLAST search [51] to identify those pairs of *M. tuberculosis* and *S. aureus* genes which were mutual best-BLAST hits of each other, called *bidirectional best-BLAST hits*. An E-value of 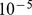 was employed as a cutoff. The second method used OrthoMCL [52] to identify pairs of genes belonging to the same ortholog group (a cross-taxa group of genes in which all genes are bidirectional best-BLAST hits of one another). The last method utilized the SEED [13] to identify genes belonging to the same FIGfam (sets of proteins homologous along their entire length).

We first identified ortholog pairs where both the *M. tuberculosis* and *S. aureus* genes were included in the *i*NJ661 and *i*SB619 models, respectively. We found that the number and content of ortholog calls depended on the method used (Figure 7A). The bidirectional best-BLAST search identified a total of 287 of a possible 619 genes. SEED identified the fewest, with only 229 orthologs. Of these, 175 orthologs were common to all methods, with smaller numbers of orthologs being shared by pairs of methods.

We also analyzed the three methods for false positive ortholog calls (Figure 7B). A false positive ortholog call is one in which two orthologs are associated with different reactions in their respective models. We found that all three methods identified 7 ortholog pairs for which model annotations were distinctly different (Table 6). SEED identified the fewest additional false positives, giving 14 total. Full details of orthologs assigned by each method can be found in Dataset S3. We then analyzed the effect of each ortholog calling method on the gene deletion sets identified by CONGA. We found that using orthologs identified by bidirectional best-BLAST and OrthoMCL yielded numerous gene deletion sets containing false positive ortholog pairs. In contrast, the number of gene deletion sets containing true ortholog pairs was relatively insensitive to the method used to call orthologs. We thus chose to perform all simulations using SEED orthologs.

**Table 6:** False positive ortholog calls in the *i*SB619 (*S. aureus*) and *i*NJ661 (*M. tuberculosis*) human pathogen models.

*S. aureus*		*M. tuberculosis*	
Locus	Reaction	Locus	Reaction
SA0486	Glutamyl-tRNA synthetase	Rv2992	Alanyl-tRNA synthetase
SA0760	Glycine cleavage complex, subunit B	Rv1826	Glycine cleavage complex, entire complex
SA1059	Methionyl-tRNA synthetase	Rv1406	Methionyl-tRNA formyltransferase
SA1131	2-oxoglutarate synthase	Rv2455	Ferredoxin oxidoreductase
SA1132	2-oxoglutarate synthase	Rv2454	Ferredoxin oxidoreductase
SA1519	L-alanine, glycine, and L-serine transport via ABC system	Rv1704	D-alanine, D-serine, glycine, and L-serine transport via proton symport
SA2467	Imidazole-glycerol-3-phosphate synthase	Rv1602	Glutamine phosphoribosyldiphosphate amidotransferase

All three methods for assigning ortholog pairs identified seven pairs of orthologs which carried out different functions in the *i*SB619 and *i*NJ661 models.

Using the orthologs identified by SEED, we then assessed the metabolic overlap between the two models (Figure 7C). In addition to the 224 orthologs present in both models, the *i*SB619 model contains 33 genes with orthologs that are not included in the *i*NJ661 model, and the *i*NJ661 model contains 13 genes with orthologs that are not in the *i*SB619 model. These 46 genes can likely be used to expand the scope of each model. Additionally, we identified 253 orthologs included in neither model. Using SEED, we were able to classify these 253 orthologs into subsystems and found that 45% were involved in protein, DNA, or RNA metabolism, while 15% were involved in non-metabolic functions such as cell division, regulation, and the stress response. An additional 22% were of unknown or uncertain function. The remaining 18% were spread across a variety of metabolic subsystems, with 35 of the 253 (8%) orthologs being involved in vitamin and cofactor synthesis. Many of these 35 genes are involved in the assembly of metal clusters and would not generally be included in a metabolic model. Finally, we observed that metabolic genes are enriched for members of an ortholog pair: 37% (229 of 619) of genes in the *i*SB619 model had orthologs in the *i*NJ661 model, while only 21% (528 of 2515) of genes in the *S. aureus* genome had orthologs in the *M. tuberculosis* genome (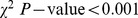).

## Discussion

In this work, we developed a bilevel mixed-integer programming approach to identify the functional differences between networks by comparing network reconstructions aligned at the gene level. The constraint-based method first identifies a set of orthologous genes based on genome sequence, and then identifies conditions under which differences in gene content give rise to differences in metabolic capabilities. Our gene-centric approach allows for the rapid identification of *functional differences* between networks which can be traced back to the presence or absence of particular genes or reactions (*structural differences*) in one network or the other. We demonstrate that our algorithm can be used to identify genetic, orthology, and metabolic differences between reaction networks with applications in metabolic engineering, model development, and antibiotic discovery.

Increasingly, new genome-scale reconstructions are being created by identifying bidirectional best-BLAST hits against genomes for which models have already been constructed. GPR and reaction annotation information can then be copied into the new model (see for example [53]–[57]). Our results point to two possible challenges with this approach. First, a bidirectional best-BLAST search might not identify all orthologs: the *i*Syp611 model was constructed from a draft *i*Cce806 model containing 591 genes. Orthologs for 537 of these genes were copied to the *i*Syp611 model, representing a 9% gene loss. Of the 54 *Cyanothece* genes for which a bidirectional best-BLAST search did not identify orthologs in *Synechococcus*, manual curation identified orthologs for 26 of them. While these orthologs were not bidirectional best-BLAST hits, we decided the genes had sufficiently high sequence similarity and sufficiently similar annotations to be considered orthologs. (Annotations were collected from NCBI, the Kyoto Encyclopedia of Genes and Genomes (KEGG) [12], and SEED [13].) This suggests that construction of new models using only bidirectional best-BLAST hits may exclude significant numbers of genes from new reconstructions. Second, using bidirectional best-BLAST hits to identify orthologs may also generate large numbers of false positive ortholog pairs. Our bidirectional best-BLAST comparison of the manually curated *S. aureus* and *M. tuberculosis* models yielded 41 false positives (14% of the 287 orthologs, where a false positive indicates orthologs were associated with different metabolic reactions). If one model had been created from the other, these genes would have incorrect reactions associated with them. Manual assessment of the cyanobacterial bidirectional best-BLAST hits yielded 35 (of 537, or 7%) false positive orthologs in the draft *i*Syp611 model, which were subsequently removed from the final reconstruction. Thus, false positive ortholog calls represent a significant problem even for closely-related organisms.

Our approach represents a significant advance in comparing genome-scale network reconstructions. CONGA is a single instance of a broader approach, in which two different networks are compared and analyzed for functional differences. This represents a significant advance over existing model-comparison approaches [7]–[9], which typically do not identify the effect of network differences on achievable functional states. However, CONGA is not a replacement for more exhaustive approaches such as jamborees or network reconciliation: CONGA will not lead to the identification of all structural differences between models, just those causing different functional states. For example, a reaction-level alignment of the *i*Syp611 and *i*Cce806 models identified 172 reactions unique to the *i*Cce806 model and 57 reactions unique to the *i*Syp611 model. Of these 229 reaction differences, 126 cannot be utilized under the photoautotrophic conditions studied here. Of the remaning 113 unique reactions, only 15 were identified by CONGA as leading to differences in gene essentiality in the two cyanobacterial models under carbon-limited photoautotrophic conditions (when all genes are considered for deletion). Additional reaction differences could be picked up by CONGA if other environments (e.g., dark fermentation), growth conditions (e.g., suboptimal instead of lethal gene deletions), and objective functions (e.g, chemical production rates) were considered, and if orphan reactions (those without a GPR association) could be deleted as well (since 20 of the 229 unique reactions did not have GPR associations). Despite the inability to identify all structural differences, CONGA can identify those gene (and thus reaction) differences which give rise to differences in predicted growth and production rates, as well as other phenotypes. As a result, we believe that it will be a useful tool to complement existing model reconciliation and comparison efforts, such as jamborees.

While this work identified gene deletions pointing to functional metabolic differences, other network perturbations may be equally effective indicators of network differences. Robust algorithms for identifying other types of perturbations have also been developed [35], [58]–[63] and can be easily incorporated into CONGA. Furthermore, gene and reaction differences may not be the only source of differences between models, differences in the representation (abstraction) of the underlying biology may also play a role. For example, the *i*AF1260 model includes a periplasmic space and an explicit (instead of lumped) representation of fatty-acid biosynthesis. Many of the reaction differences between the *i*JR904 and *i*AF1260 models arise due to these differences in model abstraction. In such a scenario, a reaction-alignment approach may be challenging, whereas using CONGA we can identify how these abstraction differences affect model predictions. CONGA can also be used to examine abstractions at the level of constraints, by comparing identical models with otherwise different constraints based on gene expression, regulation, or thermodynamics. Finally, we envision our approach being used to examine cellular behavior under different environmental conditions, or to compare evolved and un-evolved cellular phenotypes. Ultimately, a comparative approach such as ours will enable rapid evaluation of the influence of network and model differences on predicted functional states.

## Methods

### Formulation of Bilevel Optimization Problem for Identification of Gene Deletion Sets

The CONGA framework employs a bilevel optimization problem to identify genetic perturbations which disproportionately change flux through a selected reaction (e.g., growth or by-product secretion) in one organism over another (Figure 1). The outer problem is a mixed-integer linear program (MILP) which finds gene deletions maximizing the flux difference between two reactions in different models. The two inner problems (one for each model) are flux-balance analysis (FBA) problems [64], linear programs (LPs) which maximize growth subject to reaction stoichiometry, thermodynamics, and enzyme capacities. We alter the FBA problems using deletions given by the outer problem. Gene-protein-reaction (GPR) constraints associate genes with reactions and are used to enforce the gene deletions given by the outer problem. These constraints are formulated using the logical relationships developed previously [35]. CONGA can select any genes for deletion, with the restriction that orthologous genes present in both models be deleted simultaneously from both models.

The FBA formulation for each model's inner problem is shown below:
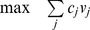
(1)

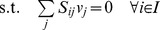
(2)

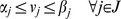
(3)

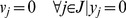
(4)


Each reaction *j* in the set of reactions *j* has a flux given by 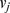. The FBA objective is a linear combination of fluxes 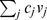, where *c* is a vector of weights. We choose to maximize for biomass alone, in which case 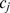 is a standard basis vector along biomass, and the objective is written as 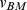. Each reaction *j* consumes and produces some metabolites *i* in the set of metabolites *I*, with stoichiometry given by 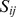. By conservation of mass, net production of each metabolite across the entire network must be zero at steady-state (equation 2). Each reaction is constrained to have flux within an appropriate range as given by enzyme capacities and thermodynamics (equation 3). For reactions deleted by the outer problem, a binary variable (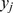) takes a zero value (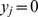), and the corresponding flux 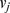 is constrained to zero (equation 4).

On-off reaction states are given by the binary variable *y* and determined by GPR constraints embedded in the outer problem:
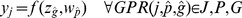
(5)


Each gene *g* in the set of genes *G*, protein *p* in the set of proteins *P*, and reaction *j* in the set of reactions *J* has a corresponding binary variable *z*, *w*, and *y*, respectively, which determines the gene, protein, or reaction's on-off state. (See [35] for details.) Each reaction *j* with a known GPR association can be carried out by a subset of enzymes 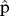, and each enzyme is specified by the subset of gene products 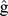. The outer problem selects one or more genes for deletion (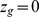), and the GPR constraints 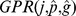 implement the necessary logical relationships to determine the set of deleted reactions (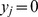).

To identify lethal gene deletion sets, the outer problem identifies deletions such that the growth rate of one species (A) is maximized with respect to the other (B). So long as growth is unconstrained, an objective of the form
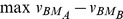
(6)will first identify gene deletions lethal only in species B. Finally, additional constraints are added which impose a limit *K* on the total number of gene deletions,
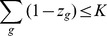
(7)and which ensure that all pairs of orthologous genes are deleted in common:
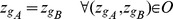
(8)The set of orthologs *O* contains all pairs of genes 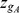 and 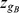 found to be orthologous between Species A and Species B.

The final formulation results from using equation (6) as the outer objective, and accumulating equations (1)–(5), (7), and (8) as constraints. Equations (1)–(5) and (7) must be imposed for each species:
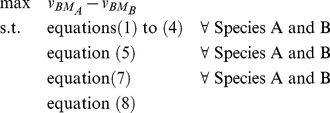



### Reformulation to Single-Level Optimization Problem

To facilitate the solution process, we reformulated the bilevel program as single-level MILP by replacing the inner maximization problems with their optimality conditions, in accordance with strong duality [65]. The strong duality theorem for a linear program states that, at optimality, the values of the primal and dual objectives are equal, and the primal and dual variables satisfy the primal and dual constraints, respectively [65]. Thus, each inner problem (equations (1) to (4)) can be replaced by formulating its dual, equating the primal and dual objectives, and accumulating the primal and dual constraints. This reformulation was first proposed for the bilevel strain design problem OptKnock [66] and has since been described for other bilevel problems [35], [60], [67].

This reformulation requires a new variable for each constraint of the inner problem. Each metabolite *i* must satisfy the mass balance, for which we introduce the unconstrained dual variable 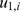. Active reactions are further constrained to be within the range 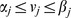, for which we introduce the positive dual variables 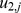 and 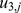, respectively. In many cases, 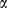 and 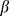 are assigned large, arbitrary values. To reduce the size of the reformulation, we eliminated the upper bound constraint (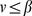) and imposed the lower bound constraint (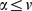) only on uptake fluxes and irreversible reactions, collectively the set 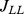. Finally, reactions removed by gene knockouts are constrained to zero flux, for which we introduce a free dual variable 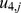. This allows the dual of each inner problem to be formulated as:
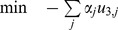
(9)

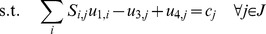
(10)

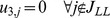
(11)

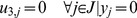
(12)

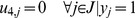
(13)

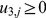
(14)


Constraints (11) to (13) can be implemented using big-M constraints [68] or using the GAMS/CPLEX indicator constraint facility (the latter was used in this work).

The single-level formulation can then be constructed by using equation (6) as the outer objective, equating the primal and dual objectives (1) and (9) for each network, including constraints (2) to (5), (7), and (10) to (14) for each network, and adding equation (8). Equating the primal and dual objectives of the inner problem gives
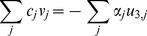
(15)so that the final, single-level formulation can be expressed as:
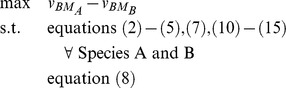



We also implemented integer cut constraints [69] to allow the generation of multiple solutions.

### Modifications for Identification of Model-Dominant Strategies

To identify model-dominant chemical production strategies in the *E. coli* models, we sought gene deletions maximizing chemical production in one model with respect to the other. For these simulations, a few modifications from the previous formulation are required. First, the outer objective, equation (6), was altered to reflect chemical production flux. The vector *c* was changed to a standard basis vector along the production flux of interest. We denote this objective as 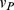.
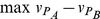
(16)


Some knockout conditions result in a *nonunique phenotype* for a particular chemical, in which multiple chemical production values can occur at the maximum growth rate. Under such conditions, CONGA can artificially inflate flux differences between models, by choosing a large production rate in one model and a small production rate in the second. We thus imposed a tilted objective function on each inner problem, which maximizes biomass while imposing a small penalty (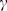) on chemical production; this causes the inner problem to return the value of 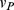 representing the lowest expected flux through the reaction [70].
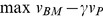
(17)


Because flux values in general are not necessarily unique, this tilted objective is necessary whenever the fluxes whose difference is being maximzed (e.g., chemical production rates) differ from the fluxes maximized by each model (e..g, biomass). We found this formulation is sensitive to the value chosen for 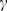. If 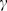 is too small, the tilt does not correctly return the value of 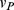 representing the lower bound through the reaction, and if 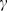 is too large, the tilt returns solutions with a slightly suboptimal growth rate. For our comparison of the *E. coli* models, we found that setting 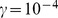 avoided both of these problems. However, the tradeoff between growth rate and chemical production flux varies from model to model and product to product, suggesting our value of 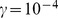 may not be generally applicable. Modifying the inner objective in this way requires modifying the weight vector *c* in equation 15 to include the value 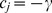 where 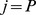. We also imposed this tilted objective function when using OptORF to identify the top deletion strategies for each model and product.

We also constrained the dual variables associated with the reaction deletion constraint to be between [−1, 1] to improve solver performance [35], [71].
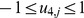
(18)


Finally, we constrained both models to have nonzero biomass. The final, single-level formulation can be expressed as:
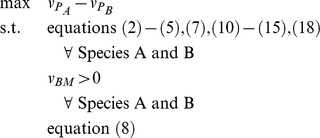



A sample implementation of CONGA for the network in Figure 2 can be found in Text S1.

Finally, we note that CONGA can be rewritten to consider a reaction alignment and reaction deletions, by redefining the set *O* (equation (8)) and using reaction instead of gene deletions (equation (7)).

### Reducing the Number of Variables

In order to reduce simulation times, we eliminated essential and blocked genes from consideration as possible deletions by CONGA. For each of the six models, we performed single-gene deletions with all possible nutrients provided to the model in order to identify essential genes (those required for cellular growth). Genes whose orthologs were essential in both models as well as essential genes without orthologs were then excluded from consideration by CONGA. Flux-variability analysis (FVA) [72] was also used, again with all possible nutrients provided, to identify blocked reactions (those incapable of carrying flux), and genes encoding only blocked reactions were also excluded from consideration (i.e., if a gene encoded both a blocked and a nonblocked reaction, CONGA was allowed to select that gene for deletion).

For our comparison of the *E. coli* models, we performed additional steps in order to improve the run-time performance of CONGA. First, we identified essential and blocked genes (as described above) on glucose media under anaerobic conditions (i.e., the simulation conditions). We also removed from consideration all genes associated with membrane transporters. These two steps forced CONGA to consider only metabolic genes that can be active under the simulation conditions. Finally, we developed a procedure to reduce the number of genes needed to determine the on-off state of each reaction. For a set of isozymes catalying the same set of reactions, all but one isozyme were fixed to the off state, such that deleting the remaining isozyme forced the set of reactions to the off state. Likewise, for a set of subunits which are all components of the same protein, all but one subunit were fixed to the on state, such that deleting the remaining subunit forced the protein to the off state. These sets of isozymes and subunits were identified using a mixed-integer programming (MIP) approach (Text S2). Finally, as in CONGA, orthologs present in both models were forced to have the same state. This ensures an internally consistent selection of isozymes and subunits (e.g., if one member of an ortholog pair is fixed to the on (or off) state, the other must be also). The complete set of isozyme and subunit sets for the two *E. coli* models can be found in Dataset S3. The same genes that were fixed to be on or off in CONGA were also fixed to be on or off in the OptORF simulations used to identify the top deletion strategies for each model and product.

### Identification of Orthologs

A gene-based alignment of two networks requires a method for identifying orthologous genes between two genomes. Since the *E. coli* simulations studied two models of the same organism, we were able to immediately match gene loci. For the cyanobacterial simulations, we used the set of bidirectional best-BLAST hits identified during the first step of the *i*Syp611 reconstruction process. Genes added during the manual reconstruction process were checked against the final *i*Cce806 model, and additional orthologs were identified. For the pathogen studies, we used SEED to identify orthologs, as this method identified the smallest number of false positive ortholog pairs (Table 6 and **Discussion**). A full summary of ortholog pairs used in each simulation can be found in Dataset S3.

### Construction of the *i*Syp611 Metabolic Network

We have formulated a genome-scale network reconstruction of the photosynthetic cyanobacterium *Synechococcus sp.* PCC 7002 consisting of 611 genes, 533 proteins, 552 reactions, and 542 metabolites (Table 7). A total of 517 reactions (94%) are associated with genes and proteins, represented by gene-protein-reaction (GPR) associations.

**Table 7:** Comparison of *i*Syp611 (*Synechococcus*) and *i*Cce806 (*Cyanothece*) cyanobacterial models.

	Draft *i*Syp611 Model	*i*Syp611 Model	*i*Cce806 Model
Genes (Orthologs)	542 (497)	611 (529)	806 (529)
Proteins	461	533	690
Reactions	491	552	667
Reactions w/GPRs	491	517	625
Metabolites	529	542	587

The draft and final reconstructions of the *i*Syp611 model differ considerably in size. The size of the *i*Cce806 model is given as a point of reference.

The model was constructed from a draft version of the *i*Cce806 reconstruction of *Cyanothece sp.* ATCC 51142 via a gene-level comparison. The *Synechococcus sp.* PCC 7002 genome sequence was downloaded from the GenBank database at the National Center for Biotechnology Information (NCBI) website [73]. A bidirectional best-BLAST search was used to identify potential orthologs between the two genomes. The validity of the associations was manually assessed using annotation information available from NCBI, KEGG, [12], and SEED [13]. For those genes deemed highly probable orthologs, protein and reaction associations were copied from the *i*Cce806 model to create a draft reconstruction using SimPheny (Genomatica Inc., San Diego, CA).

After assembling the draft network, missing functions were added to ensure production of all biomass components. Candidate reactions were selected based on pathways in other cyanobacterial strains. Potential genes for these reactions were located by best-hit BLAST analysis against other cyanobacterial genomes as well as annotation information obtained from NCBI, KEGG, and SEED. In cases where genomic information was unavailable, reactions were selected based on their frequency of occurrence in related strains. This draft model contained 542 genes, of which 497 were orthologous to genes in *Cyanothece* (Table 7).

We also applied CONGA to our draft reconstruction, and use the results to add new subunits and isozymes to existing reactions. In all, nearly 70 genes were added to the reconstruction. A complete list of reactions and GPR associations in the *i*Syp611 model is included in the Supporting Information, in Microsoft Excel and SBML formats (Datasets S1 and S2).

Wherever possible, the reactions used to represent RNA, DNA, protein, fatty acid, and lipid synthesis were updated to reflect the particulars of *Synechococcus sp.* PCC 7002. DNA and RNA composition was based on genomic GC content, and protein composition was obtained from amino acid counts of the proteome. Fatty acid composition was taken from *Synechococcus sp.* PCC 7002 [74], [75], and lipid composition was taken from *Synechococcus sp.* PCC 7942 [76]. The biomass equation was formulated using weight fractions of macromolecules (DNA, RNA, protein, lipid, fatty acids, glycogen) measured from *Synechosystis sp.* PCC 6803 in batch culture [77], and the composition of the soluble pool was copied from the *i*JR904 model [15].

The final metabolic reconstruction was used to formulate a constraint-based model of *Synechococcus* metabolism. Experimentally, *Synechococcus* is able to grow phototrophically using light, carbon dioxide, and ammonium. Our constraint-based model was capable of predicting growth under photoautotrophic and glycerol heterotrophic conditions via FBA.

### Models and Simulation Conditions

The CONGA analysis of the two published models of *E. coli*, *i*JR904 [15] and *i*AF1260 [16] were performed under anaerobic, glucose-limited conditions (uptake rate 18.5 mmol/gDW/hr). All reported chemical production levels were normalized to the theoretical maximum (2 mol ethanol/mol glucose, 2 mol lactate/mol glucose, and 1.71 mol succinate/mol glucose).

For the *i*Syp611 and *i*Cce806 comparisons, several reactions in the *i*Syp611 model were replaced with their *i*Cce806 equivalents, including biomass, ATP synthase, DNA, RNA, lipid, and protein synthesis, and cytochrome oxidases unique to *Cyanothece* (Dataset S2). Simulations were performed under carbon-limited photoautotrophic conditions, with maximum uptake fluxes for photons for both photosystems, carbon dioxide, and ammonium constrained to 100 mmol photons/gDW/hr, 20 mmol/gDW/hr, and 10 mmol/gDW/hr, respectively. Unconstrained uptake of inorganic phosphate, oxygen, magnesium(II), protons, sulfate, and water was also allowed. Growth-associated and non-growth associated ATP maintenance requirements were set to zero.

For the *i*NJ661 and *i*SB619 models, simulations were performed on nine distinct minimal media with different carbon and nitrogen sources (Dataset S2). The set of gene deletion sets common to all conditions was then analyzed for potential drug targets.

All simulations were performed using CPLEX 12 (IBM, Armonk, NY) accessed via the General Algebraic Modeling System (GAMS, GAMS Development Corporation, Washington, DC). Simulations were performed on a Red Hat Enterprise Linux server with 2.66 GHz Intel Xeon processors and 8 GB of RAM. CONGA can identify a lethal gene deletion set containing just one gene in less than a second. Lethal gene deletion sets containing two or three genes took on average 3 minutes to identify. Identifying model-dominant chemical production strategies is more time-consuming, with a model-dominant strategy containing two genes requiring on average 2 minutes. Model-dominant strategies containing three, four, or five genes took an average of 15 minutes, 75 minutes, and 5 hours to identify, respectively. A full summary of differences identified by each simulation can be found in Dataset S3.

## Supporting Information

Figure S1 **Model-dominant production strategies for lactate.** (**A**) Deletion strategies for lactate production. Each bar represents the absolute difference in predicted lactate yields between the *i*JR904 and *i*AF1260 models as a fraction of the maximum theoretical yield (2 lactate/glucose). Left side: Strategies for which the *i*AF1260 model predicts higher production. Right side: Strategies for which the *i*JR904 model predicts higher production. Corresponding gene deletion strategies involving 3, 4, or 5 genes are given below the figure. Numbers above each bar indicate the fraction of the theoretical maximum yield obtained by each model, with the dominant model listed first. Some strategies have a nonunique lactate production phenotype, in which multiple lactate production values can occur at the maximum growth rate. For these scenarios, the production difference calculated by CONGA is from the lowest expected level of lactate production in each model, and such strategies are indicated in green. Strategies for which the yield of the dominant model meets or exceeds the yield for the third-best OptORF strategy for that model are known as OptORF strategies, and such strategies are indicated in red. OptORF strategies which also have a nonunique production phenotype are indicated in orange. (**B**) The same gene deletion strategies after reconciliation of the *i*JR904 and *i*AF1260 networks with respect to metabolic differences.
(PDF)

Figure S2 **Model-dominant production strategies for succinate.** (**A**) Deletion strategies for succinate production. Each bar represents the absolute difference in predicted succinate yields between the *i*JR904 and *i*AF1260 models as a fraction of the maximum theoretical yield (1.71 succinate/glucose). Left side: Strategies for which the *i*AF1260 model predicts higher production. Right side: Strategies for which the *i*JR904 model predicts higher production. Corresponding gene deletion strategies involving 2, 3, or 4 genes are given below the figure. Genes enclosed in parentheses indicate a deletion unique to the *i*AF1260 model. Numbers above each bar indicate the fraction of the theoretical maximum yield obtained by each model. Strategies for which the yield of the dominant model meets or exceeds the yield for the third-best OptORF strategy for that model are known as OptORF strategies, and such strategies are indicated in red. (**B**) The same gene deletion strategies after reconciliation of the *i*JR904 and *i*AF1260 networks with respect to metabolic differences.
(PDF)

Figure S3
**Differences in metabolic pathways in** ***E. coli*** **GENREs.** The text above each map indicates the pathway responsible for the phenotypic difference, the phenotype with which the strategy is associated, and the gene deletion for which the phenotype occurs. (**A**–**C**) Schematic views of the flux distributions associated with the indicated gene deletion set. Metabolites are represented in plain text. Metabolites are represented in plain text. Metabolic transformations are indicated via arrows, with thicker arrows indicating higher flux. In some instances, multiple transformations are combined into a single dashed arrow. Fluxes active in the *i*AF1260 network are in red, fluxes active in the *i*JR904 network are in blue, and inactive fluxes are in grey. If gene (reaction) deletions occur in the fermentation pathway, they are indicated by black ‘X’s. Fluxes crossing the dashed boundary indicate transport to the extracellular environment. Metabolite abbreviations: 2aobut, L-2-Amino-3-oxobutanoate; actp, acetyl phosphate; athr, allo-threonine; glc, glucose. All other abbreviations match those used in the *i*Syp611 metabolic model (see Additional File 2). (**A**) As a consequence of growth, *E. coli* produces protons which must be consumed by reactions in the network or secreted into the media. The succinate (succ) antiporter in the *i*AF1260 model makes succinate transport energetically unfavorable, so flux is instead diverted to ethanol (etoh). (**B**) This knockout eliminates ATP synthase, necessary for pumping protons into the cytosol. Deletion of ATP synthase forces protons to be consumed via the fermentation reactions. The ability of the *i*AF1260 model to transport 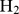 allows it to utilize formate hydrogen lyase for this purpose. As a consequence, flux is diverted away from ethanol to 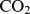. This effect becomes more pronounced as additional genes are deleted. (**C**) Wild-type FBA predicts that both *E. coli* models uptake glucose via the phosphotransferase system (PTS), and convert the resulting glucose-6-phosphate (g6p) to fructose-6-phosphate (f6p). The deletion of *pgi* prevents this transformation. The *i*AF1260 network contains an additional hexokinase which allows it to bypass the deletion by bypassing the PTS. The *i*JR904 model lacks this reaction and is forced to use PTS. The deletion of *edd* then forces the *i*JR904 network to metabolize glucose via the oxidative branch of the pentose phosphate pathway. This process generates electrons, leading to higher levels of fermentation products than in the *i*AF1260 model.(PDF)

Dataset S1 **SBML version of the** ***i*** **Syp611 model.**
(XML)

Dataset S2 **Microsoft Excel versions of the** ***i*** **Syp611 model.** Description of stoichiometric and annotation changes made to each of the six metabolic models examined over the course of this study.
(XLSX)

Dataset S3 **Computational results of all simulations.** This Microsoft Excel file contains 1) the simulation conditions for each study, 2) the full results of all CONGA simulations performed in this study, 3) all ortholog assignments used in performing the CONGA simulations, and 4) results from each ortholog calling method applied to the human pathogen models.
(XLSX)

Text S1 **Sample implementation of CONGA formulation in GAMS format.** A free demo version of GAMS can be downloaded from http://www.gams.com. This file contains the example network described in Figure 2.
(GMS)

Text S2 **Variable Reduction Procedure.** This section contains additional details about the algorithm used to decrease the number of genes considered by CONGA.
(PDF)
