# Supplementary material for: Identification of Functional Differences in Metabolic Networks Using Comparative Genomics and Constraint-Based Models
Source: PLoS One. 2012 Apr 16;7(4):e34670. doi: 10.1371/journal.pone.0034670 (PMC3359066; doi:10.1371/journal.pone.0034670)
Supplement: Figure S1 — Model-dominant production strategies for lactate. (A) Deletion strategies for lactate production. Each bar represents the absolute difference in predicted lactate yields between the iJR904 and iAF1260 models as a fraction of the maximum theoretical yield (2 lactate/glucose). Left side: Strategies for which the iAF1260 model predicts higher production. Right side: Strategies for which the iJR904 model predicts higher production. Corresponding gene deletion strategies involving 3, 4, or 5 genes are given below the figure. Numbers above each bar indicate the fraction of the theoretical maximum yield obtained by each model, with the dominant model listed first. Some strategies have a nonunique lactate production phenotype, in which multiple lactate production values can occur at the maximum growth rate. For these scenarios, the production difference calculated by CONGA is from the lowest expected level of lactate production in each model, and such strategies are indicated in green. Strategies for which the yield of the dominant model meets or exceeds the yield for the third-best OptORF strategy for that model are known as OptORF strategies, and such strategies are indicated in red. OptORF strategies which also have a nonunique production phenotype are indicated in orange. (B) The same gene deletion strategies after reconciliation of the iJR904 and iAF1260 networks with respect to metabolic differences. (PDF) [file pone.0034670.s001.pdf]

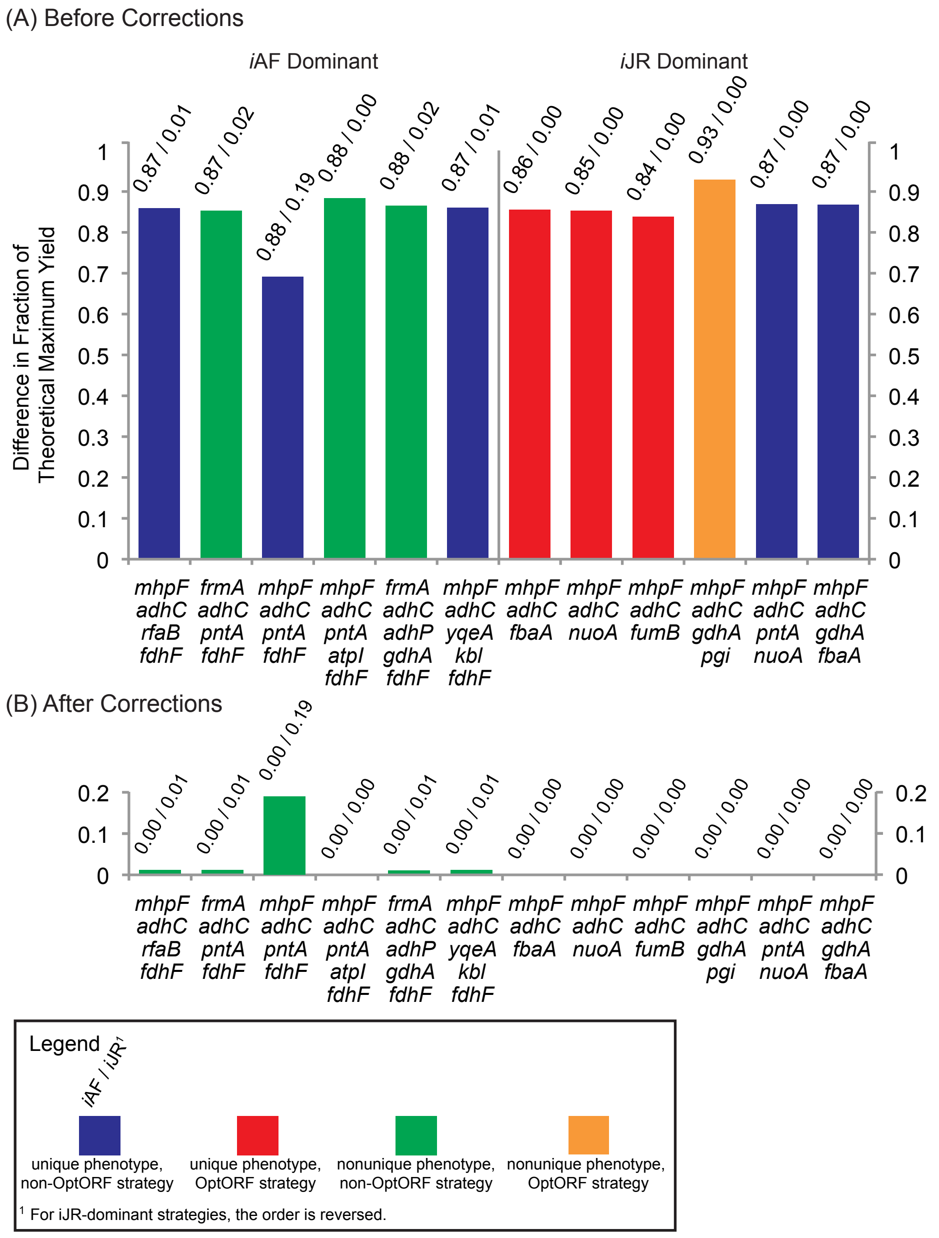
(A) Before Corrections
iAF Dominant
iJR Dominant
Difference in Fraction of Theoretical Maximum Yield
0.87 / 0.01
0.87 / 0.02
0.88 / 0.19
0.88 / 0.00
0.88 / 0.02
0.87 / 0.01
0.86 / 0.00
0.85 / 0.00
0.84 / 0.00
0.93 / 0.00
0.87 / 0.00
0.87 / 0.00
0 0.1 0.2 0.3 0.4 0.5 0.6 0.7 0.8 0.9 1
mhpF adhC rfaB fdhF
frmA adhC pntA fdhF
mhpF adhC pntA fdhF
mhpF adhC pntA atpI fdhF
frmA adhC adhP gdhA fdhF
mhpF adhC yqeA kbl fdhF
mhpF adhC fbaA
mhpF adhC nuoA
mhpF adhC fumB
mhpF adhC gdhA pgi
mhpF adhC pntA nuoA
mhpF adhC gdhA fbaA
(B) After Corrections
0.00 / 0.01
0.00 / 0.01
0.00 / 0.19
0.00 / 0.00
0.00 / 0.01
0.00 / 0.01
0.00 / 0.00
0.00 / 0.00
0.00 / 0.00
0.00 / 0.00
0.00 / 0.00
0.00 / 0.00
0 0.1 0.2
mhpF adhC rfaB fdhF
frmA adhC pntA fdhF
mhpF adhC pntA fdhF
mhpF adhC pntA atpI fdhF
frmA adhC adhP gdhA fdhF
mhpF adhC yqeA kbl fdhF
mhpF adhC fbaA
mhpF adhC nuoA
mhpF adhC fumB
mhpF adhC gdhA pgi
mhpF adhC pntA nuoA
mhpF adhC gdhA fbaA
Legend
iAF / iJR[1]
unique phenotype, non-OptORF strategy
unique phenotype, OptORF strategy
nonunique phenotype, non-OptORF strategy
nonunique phenotype, OptORF strategy
[1] For iJR-dominant strategies, the order is reversed.
