# Supplementary material for: Identification of Functional Differences in Metabolic Networks Using Comparative Genomics and Constraint-Based Models
Source: PLoS One. 2012 Apr 16;7(4):e34670. doi: 10.1371/journal.pone.0034670 (PMC3359066; doi:10.1371/journal.pone.0034670)
Supplement: Figure S2 — Model-dominant production strategies for succinate. (A) Deletion strategies for succinate production. Each bar represents the absolute difference in predicted succinate yields between the iJR904 and iAF1260 models as a fraction of the maximum theoretical yield (1.71 succinate/glucose). Left side: Strategies for which the iAF1260 model predicts higher production. Right side: Strategies for which the iJR904 model predicts higher production. Corresponding gene deletion strategies involving 2, 3, or 4 genes are given below the figure. Genes enclosed in parentheses indicate a deletion unique to the iAF1260 model. Numbers above each bar indicate the fraction of the theoretical maximum yield obtained by each model. Strategies for which the yield of the dominant model meets or exceeds the yield for the third-best OptORF strategy for that model are known as OptORF strategies, and such strategies are indicated in red. (B) The same gene deletion strategies after reconciliation of the iJR904 and iAF1260 networks with respect to metabolic differences. (PDF) [file pone.0034670.s002.pdf]

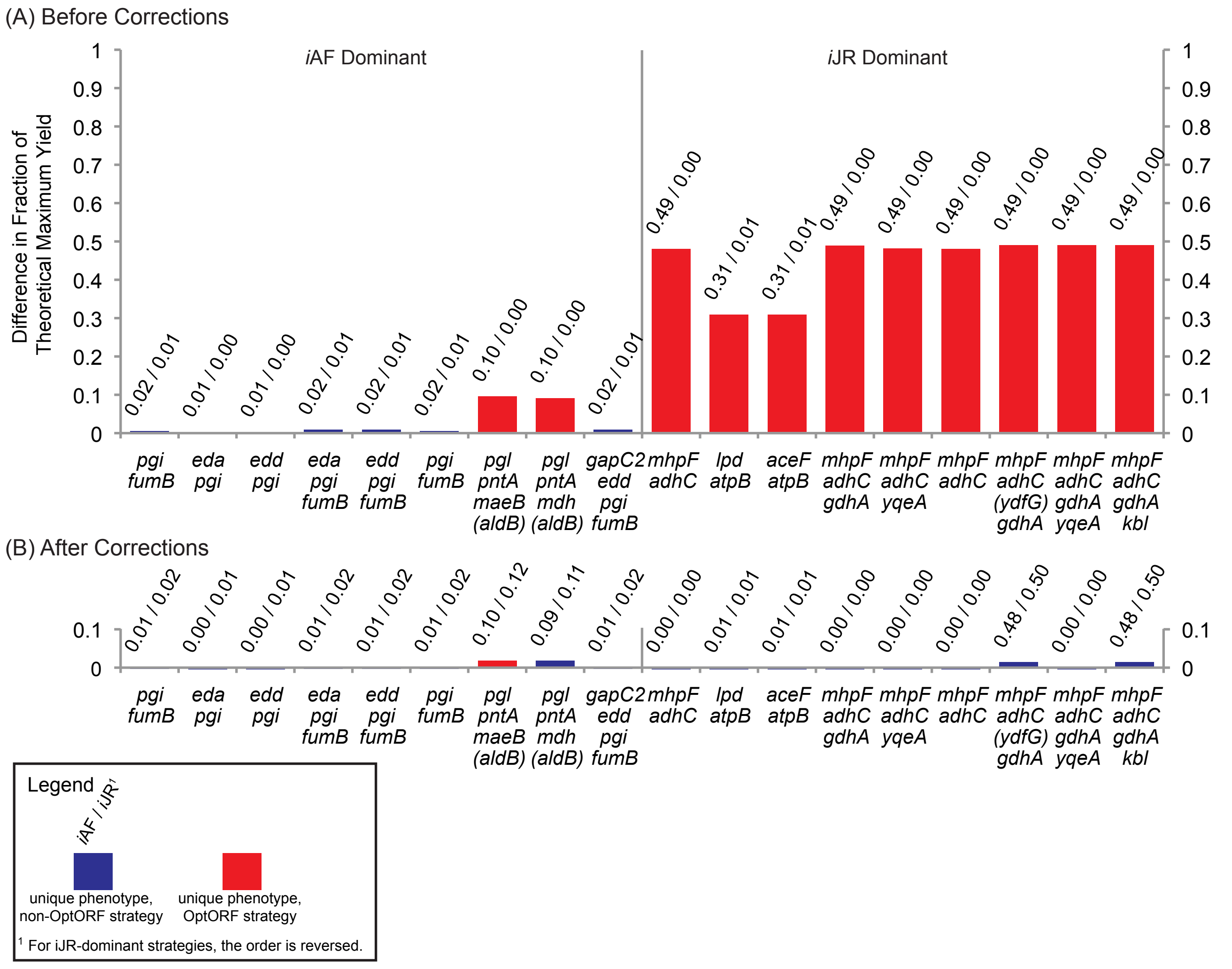
(A) Before Corrections
iAF Dominant
iJR Dominant
Difference in Fraction of Theoretical Maximum Yield
0.02 / 0.01
0.01 / 0.00
0.01 / 0.00
0.02 / 0.01
0.02 / 0.01
0.02 / 0.01
0.10 / 0.00
0.10 / 0.00
0.02 / 0.01
0.49 / 0.00
0.31 / 0.01
0.31 / 0.01
0.49 / 0.00
0.49 / 0.00
0.49 / 0.00
0.49 / 0.00
0.49 / 0.00
0.49 / 0.00
pgi fumB
eda pgi
edd pgi
eda pgi fumB
edd pgi fumB
pgi fumB
pgl pntA maeB (aldB)
pgl pntA mdh (aldB)
gapC2 edd pgi fumB
mhpF adhC
lpd atpB
aceF atpB
mhpF adhC gdhA
mhpF adhC yqeA
mhpF adhC
mhpF adhC (ydfG) gdhA
mhpF adhC gdhA yqeA
mhpF adhC gdhA kbl
(B) After Corrections
0.01 / 0.02
0.00 / 0.01
0.00 / 0.01
0.01 / 0.02
0.01 / 0.02
0.01 / 0.02
0.10 / 0.12
0.09 / 0.11
0.01 / 0.02
0.00 / 0.00
0.01 / 0.01
0.01 / 0.01
0.00 / 0.00
0.00 / 0.00
0.00 / 0.00
0.48 / 0.50
0.00 / 0.00
0.48 / 0.50
Legend
iAF / iJR[1]
unique phenotype, non-OptORF strategy
unique phenotype, OptORF strategy
[1] For iJR-dominant strategies, the order is reversed.
