# Supplementary material for: Identification of Functional Differences in Metabolic Networks Using Comparative Genomics and Constraint-Based Models
Source: PLoS One. 2012 Apr 16;7(4):e34670. doi: 10.1371/journal.pone.0034670 (PMC3359066; doi:10.1371/journal.pone.0034670)

(A) Role of Succinate Transport in *i*AF Dominant Ethanol Production.
Deletions: *ycgT*, dihydroxyacetone phosphotransferase
pykA, pyruvate kinase

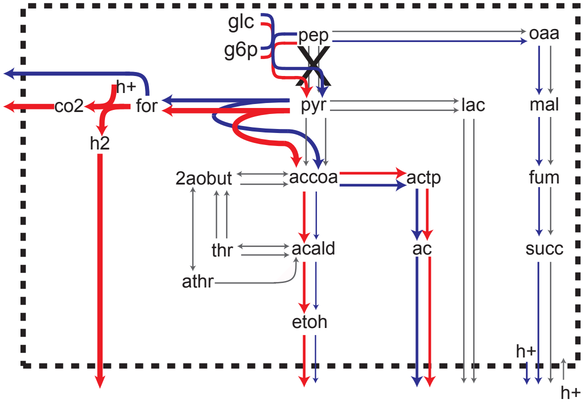

(B) Role of Hydrogen Transport in *i*JR Dominant Ethanol Production.
Deletions: *atpB*, ATP synthase

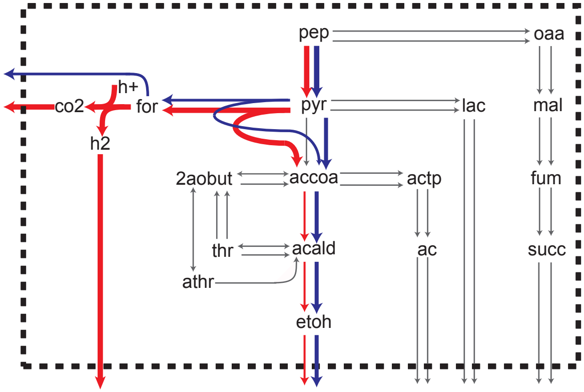

(C) Role of Hexokinase in *i*JR Dominant Ethanol Production.
Deletions: *edd*, 6-phosphogluconate dehydratase
pgi, glucose-6-phosphate isomerase

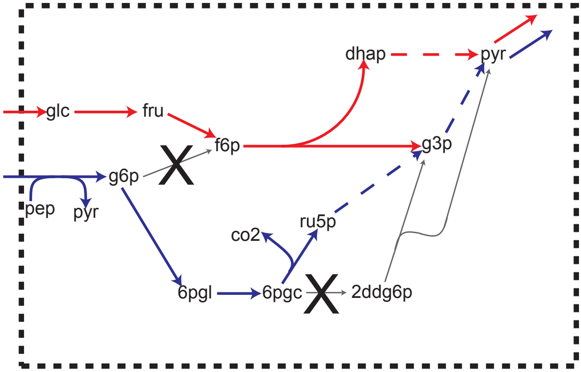

Supplement: Figure S3 — Differences in metabolic pathways in E. coli GENREs. The text above each map indicates the pathway responsible for the phenotypic difference, the phenotype with which the strategy is associated, and the gene deletion for which the phenotype occurs. (A–C) Schematic views of the flux distributions associated with the indicated gene deletion set. Metabolites are represented in plain text. Metabolites are represented in plain text. Metabolic transformations are indicated via arrows, with thicker arrows indicating higher flux. In some instances, multiple transformations are combined into a single dashed arrow. Fluxes active in the iAF1260 network are in red, fluxes active in the iJR904 network are in blue, and inactive fluxes are in grey. If gene (reaction) deletions occur in the fermentation pathway, they are indicated by black ‘X’s. Fluxes crossing the dashed boundary indicate transport to the extracellular environment. Metabolite abbreviations: 2aobut, L-2-Amino-3-oxobutanoate; actp, acetyl phosphate; athr, allo-threonine; glc, glucose. All other abbreviations match those used in the iSyp611 metabolic model (see Additional File 2). (A) As a consequence of growth, E. coli produces protons which must be consumed by reactions in the network or secreted into the media. The succinate (succ) antiporter in the iAF1260 model makes succinate transport energetically unfavorable, so flux is instead diverted to ethanol (etoh). (B) This knockout eliminates ATP synthase, necessary for pumping protons into the cytosol. Deletion of ATP synthase forces protons to be consumed via the fermentation reactions. The ability of the iAF1260 model to transport allows it to utilize formate hydrogen lyase for this purpose. As a consequence, flux is diverted away from ethanol to . This effect becomes more pronounced as additional genes are deleted. (C) Wild-type FBA predicts that both E. coli models uptake glucose via the phosphotransferase system (PTS), and convert the resulting glucose-6-phosph [file pone.0034670.s003.pdf]
