# Supplementary material for: Identification of Functional Differences in Metabolic Networks Using Comparative Genomics and Constraint-Based Models
Source: PLoS One. 2012 Apr 16;7(4):e34670. doi: 10.1371/journal.pone.0034670 (PMC3359066; doi:10.1371/journal.pone.0034670)
Supplement: Text S2 — Variable Reduction Procedure. This section contains additional details about the algorithm used to decrease the number of genes considered by CONGA. (PDF) [file pone.0034670.s008.pdf]

Reducing the Number of Variables

1 Operations Performed on All Models

In order to reduce simulation times, we eliminated essential and blocked genes from consideration as possible deletions by CONGA. For each model, we performed single-gene deletion simulations with no constraints on uptake fluxes to identify essential genes (those required for cellular growth). Genes whose orthologs were essential in both models as well as essential genes without orthologs (collectively the set G_E) were then excluded from consideration by CONGA. Eliminating single-deletion essential genes from consideration enables CONGA to focus only on conditionally essential genes, as well as those deletions for which exhaustive searches of all combinations are computationally prohibitive.

Flux-variability analysis (FVA) was also used to identify blocked reactions (those incapable of carrying flux), and genes encoding only blocked reactions were also identified (e.g., if a gene encodes both a blocked and a nonblocked reaction, the gene is not considered a blocked gene). As above, genes whose orthologs were blocked in both models as well as blocked genes without orthologs (collectively the set G_B) were then excluded from consideration by CONGA. Because blocked reactions cannot cary flux under any circumstances, we know *a priori* that blocked genes cannot contribute to functional network differences.

For each pair of models, we fixed the essential genes of each organism to be on ($z_g = 1$), thereby excluding them from consideration by CONGA. We also fixed the blocked genes of each organism to be off ($z_g = 0$), and excluded them from the gene-deletion constraint, $\sum_g (1 - z_g) \leq K$. The remaining genes which do not have a fixed on-off state make up the selectable gene set, G_S (Table S1). For our *E. coli* comparisons, the sets G_E and G_B were identified on glucose media under anerobic conditions (i.e., the simulation conditions). Because these simulations enforced a nonzero biomass requirement, essential genes cannot play a role in model-dominant gene deletion strategies. We also added all genes encoding transport reactions were also added G_E, allowing us to focus on enzymatic, rather than transport, reaction differences between the networks.

2 Additional Operations for *E. coli* Models

We found that CONGA was slow to identify model-dominant strategies in the *E. coli* models when only essential and blocked genes were eliminated, due to the large size of the models. To further reduce the number of genes CONGA had available for consideration (and thereby to improve run-time performance), we developed a procedure to reduce the number of genes needed to determine the on-off state of each reaction, by identifying conserved sets of subunits and isozymes across models.

We first constructed and solved an optimization problem to determine the reactions which can be activated by each gene $\bar{g}$ in the model: we turn off all genes but $\bar{g}$, and identify those reactions which can be turned on ($y_j = 1$) by activating $\bar{g}$ alone, subject to GPR constraints. We refer to the set of such reactions as the the *activated reaction set*; genes with the same activated reaction set correspond to isozymes.

$$\begin{array}{llll} \max & \sum_j y_j & & \\ & z_{\bar{g}} = 1 & & \\ & z_g = 0 & \forall\ g & \in G \setminus \bar{g} \\ & y_j = f(z_{\hat{g}}, w_{\hat{p}}) & \forall\ \mathrm{GPR}(j, \hat{p}, \hat{g}) & \in J, P, G \end{array}$$

We then constructed and solved an optimization problem to determine the reactions which can be deactivated by each gene $\bar{g}$ in the model: we turn on all genes but $\bar{g}$, and find those reactions which can be turned off ($y_j = 0$) by deleting $\bar{g}$ alone, subject to GPR constraints. We refer to the set of such reactions as the the *deactivated reaction set*; genes with the same deactivated reaction set correspond to subunits (or

members of the same protein complex).

$$
\begin{aligned}
\max \quad & \sum_j y_j & & \\
& z_{\bar{g}} = 0 & & \\
& z_g = 1 & \forall\ g & \in G \setminus \bar{g} \\
& y_j = f(z_{\hat{g}}, w_{\hat{p}}) & \forall\ \text{GPR}(j, \hat{p}, \hat{g}) & \in J, P, G
\end{aligned}
$$

We then identified all isozymes and subunits (called *gene sets*) by identifying the sets of genes which all have the same activated or deactivated reaction sets. Some isozymes or subunits within a gene set may be multi-functional (e.g., be associated with multiple reactions); these are included in the gene set only if all isozymes or subunits within the set share the same multi-functionality. We then manually aligned the sets of isozymes and subunits between the models. We find that subunits and isozymes can align in different ways, as illustrated in Figure S4 on the next page.

In scenario 1, the gene sets are identical in both models. (Alternatively, the genes in a gene set are only found in one model.) In this case, we select a single gene to represent the state of the entire gene set. We call this gene the *selectable gene*. In scenario 2, gene sets in the two models partially overlap, with each set containing both conserved and unique genes. In this case, we select a single gene to represent the state of the conserved genes, and a single gene to represent the unique portion of each gene set, for a total of three selectable genes. And finally in scenario 3, a gene set in one model overlaps with multiple gene sets in the second model. In this case, each individual gene remains selectable. Many additional clarifying examples can be found can be found in the Supporting Information (Dataset S3).

For each gene set corresponding to a group of isozymes, we fix all but the selectable gene from each set to the off state, $z_g = 0$ (collectively the "off set", G_{off}). For a reaction with isozymes, this procedure ensures that all but one common isozyme is fixed to the off state, so that deleting selectable isozymes forces the reactions to the off state. For each gene set corresponding to a group of subunits, we fix all but the selectable gene from each set to the on state, $z_g = 1$ (collectively the "on set", G_{on}). For a protein with subunits, this procedure ensures that all but one common subunit is fixed to the on state, so that deleting selectable subunits forces reactions to the off state. This procedure also prevents equivalent solutions from being found by CONGA (e.g., multiple gene deletion sets corresponding to the same reaction deletion set). The full list of gene sets in the *i*JR904 and *i*AF1260 models can be found in the Supporting Information (Dataset S3).

The genes fixed on or off by the above two procedures (referred to as redundant genes in Table S1) were removed from the selectable gene set, G_S. The new, smaller selectable gene set represents a subset set of genes which can determine the on-off state of all non-essential, non-blocked reactions in a pair of models (Table S1). With these new constraints, the bilevel form of CONGA can be written as:

$$
\begin{aligned}
\max \quad & \text{difference in flux} & & & \\
& \text{equations (1) to (4)} & & & \forall\ \text{Species A and B} \\
& \text{equation (5)} & & & \forall\ \text{Species A and B} \\
& \sum_{g \in G_S} (1 - z_g) \leq K & & & \forall\ \text{Species A and B} \\
& z_g = 1 & \forall\ g & \in G_E \text{ and } G_{on} & \forall\ \text{Species A and B} \\
& z_g = 0 & \forall\ g & \in G_B \text{ and } G_{off} & \forall\ \text{Species A and B} \\
& \text{equation (8)} & & &
\end{aligned}
$$

(Refer to the manuscript for numbered equations.)

Figure S4: Alignment of isozymes and subunits. We find that subunits and isozymes can align in different ways, as illustrated below. See the main text for a description of each pattern.

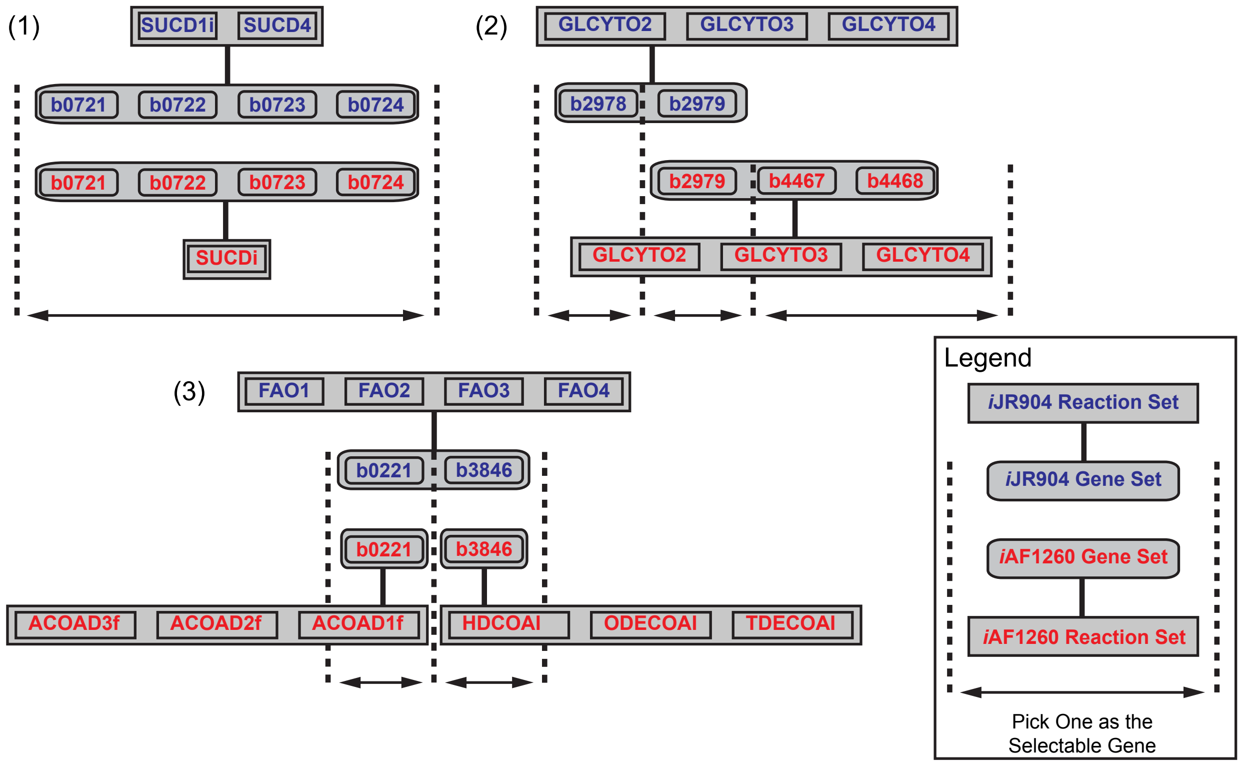

Table S1: Variable Reduction Procedures. Numbers of genes eliminated via identification of essential, blocked, and redundant genes in each of the six models studied. The final number of genes left for CONGA to select from is also given. A solid black line indicates the procedure was not carried out on the indicated model.

Model	Size[1]	Essential	Blocked	Redundant	Selectable	% Original Size
*i*JR904	905	171	171	168	395	44
*i*AF1260	1260	215	279	253	523	42
*i*Cce806	806	214	178	—	414	51
*i*Syp611	611	254	131	—	226	37
*i*SB619	615	80	142	—	393	64
*i*NJ661	655	128	107	—	420	64

[1] The size of the model represents the number of genes after the initial model reconciliation steps.
